# Habitat Specialisation Impacts Clownfish Demographic Resilience to Pleistocene Sea‐Level Fluctuations

**DOI:** 10.1111/mec.70134

**Published:** 2025-10-13

**Authors:** Alberto García‐Jiménez, Marion Talbi, Lucy M. Fitzgerald, A. Heim, Anna Marcionetti, Sarah Schmid, Joris Bertrand, Abigail Shaughnessy, Carl Santiago, Ploypallin Rangseethampanya, Phurinat Ruttanachuchote, Wiphawan Aunkhongthong, Sittiporn Pengsakun, Makamas Sutthacheep, Milan Malinsky, Bruno Frédérich, Fabio Cortesi, Marc Kochzius, Thamasak Yeemin, Théo Gaboriau, Nicolas Salamin

**Affiliations:** ^1^ Department of Computational Biology University of Lausanne Lausanne Switzerland; ^2^ Department of Aquatic Ecology & Evolution University of Bern Bern Switzerland; ^3^ Department of Environmental Systems Science ETH Zürich Zürich Switzerland; ^4^ Laboratoire Génome et Développement des Plantes UPVD Perpignan France; ^5^ Queensland Brain Institute The University of Queensland Brisbane Australia; ^6^ Marine Biodiversity Research Group Ramkhamhaeng University Bangkok Thailand; ^7^ Laboratory of Evolutionary Ecology FOCUS, University of Liège Liège Belgium; ^8^ Marine Biology‐Ecology, Evolution and Genetics (bDIV) Vrije Universiteit (VUB) Brussels Belgium

**Keywords:** clownfish mutualism, demographic resilience, genetic structure, habitat specialisation, population genomics, sea‐level fluctuations

## Abstract

Habitat fragmentation and loss are key threats to biodiversity, yet their impacts on marine species remain poorly understood. Clownfishes, which rely on sea anemones for shelter and reproduction, provide an interesting model to explore how ecological specialisation mediates species responses to habitat perturbations. We used whole‐genome data from 382 individuals across 10 species with varying host specialisations to reconstruct demographic histories and infer spatial genetic structure to assess the impact of Pleistocene sea‐level fluctuations. Generalist species, associated with multiple hosts, maintained stable effective population sizes ($N_{e}$) and population connectivity during habitat fragmentation, reflecting resilience to environmental instability. In contrast, specialists experienced severe $N_{e}$ declines and genetic structuring, driven by their dependence on specific hosts, without signs of population recovery following habitat reconnection. Spatial genomic analyses identified the Indonesian Through‐Flow as a key dispersal corridor and the Coral Triangle as a critical hub of genetic diversity, while continental shelves and extensive open ocean regions appeared as barriers to gene flow. Our findings reveal how host specialisation shapes clownfish population dynamics, emphasising the importance of incorporating ecological dependencies into conservation assessments and deepening our understanding of species responses to ecological constraints and environmental changes over evolutionary timescales.

## 1 Introduction

Habitat loss and fragmentation are major threats to biodiversity and ecosystem stability (Crooks et al. 2017). Although habitat loss has consistently negative effects (Fahrig 2003; Crespo‐Miguel and Jarillo 2022), the impacts of fragmentation are context‐dependent, varying with fragmentation scale, species traits and habitat configuration (Ibáñez et al. 2014; Haddad et al. 2015; Fahrig 2017). The effects of fragmentation are well studied in terrestrial systems, where fragmentation often promotes isolation and population divergence, potentially fostering speciation (Tolley et al. 2011; Habel and Zachos 2012) but also causes genetic bottlenecks and loss of genetic diversity (Habel and Zachos 2012; Young et al. 2018). In contrast, the effects of large‐scale fragmentation on marine organisms, in particular reef‐associated species, remain limited due to challenges in studying connectivity and gene flow in open ocean environments (Fraser et al. 2014).

Sea‐level fluctuations throughout the Quaternary (∼2.6 million to 11,700 years ago) profoundly reshaped coral reef ecosystems, fragmenting habitats and altering species distributions and connectivity (Pellissier et al. 2014). During glacial periods, sea‐level drops exposed continental shelves, leading to widespread habitat loss and reproductive isolation among reef‐associated species (Fauvelot et al. 2003; Ludt and Rocha 2015). These fluctuations would have promoted population divergence, fostering the emergence of distinct genetic lineages and endemic species within isolated reef systems (Bowen et al. 2013; Cowman et al. 2017). However, Delrieu‐Trottin et al. (2020) documented expansions in endemic reef fish species around Easter Island, illustrating both the opportunities and constraints imposed by such habitat perturbations. While some species adapted and diversified in response to Quaternary sea‐level fluctuations, others experienced reduced genetic diversity and population declines due to limited dispersal and ecological specialisation (Gaither et al. 2015). Understanding how these historical processes have shaped marine biodiversity remains a central challenge, especially for taxa with obligate mutualisms or reliant on highly specialised habitats, where the impacts of fragmentation were likely most pronounced.

Among reef fishes, clownfishes (subfamily Amphiprioninae) offer an ideal model for studying the effects of historical fragmentation and ecological specialisation on population dynamics. Clownfishes rely on a unique mutualistic relationship with sea anemones for shelter and reproduction (Fautin 1991), with no observations of clownfishes living independently of sea anemones (Elliott et al. 1995; Buston 2003). This ecological dependence on specific hosts has profound implications for clownfish resilience to habitat changes (Salles et al. 2020), with clownfish distributions intrinsically tied to those of their hosts (García Jiménez et al. 2024). Coupled with a short pelagic larval dispersal stage, lasting approximately 15 days and a limited dispersal range (Pinsky et al. 2010; Berumen et al. 2012), this dependence on hosts highlights the constraints on the ability of clownfish to colonise new habitats, making them particularly susceptible to habitat fragmentation and loss. Clownfish species vary considerably in their host specificity, with some forming exclusive associations with particular anemones, such as *Radianthus magnifica*, *Entacmaea quadricolor* or members of the *Stichodactyla* genus, while others exhibit generalist behaviour, capable of associating with multiple anemone species (Gaboriau et al. 2025). This generalist‐specialist axis provides a valuable framework for examining the impact of specialisation on population structure, resilience and vulnerability to habitat fragmentation.

Despite the ecological and evolutionary importance of the mutualism between clownfish and sea anemones, relatively little is known about the role that historical habitat fragmentation plays in their population histories. Previous genetic studies on reef fish have been limited by markers lacking the fine‐scale resolution needed to capture landscape‐level patterns and species‐specific responses to fragmentation (Hellberg 2009). However, advances in population genomics now allow for detailed reconstructions of demographic histories, allowing researchers to examine how historical processes such as Pleistocene sea‐level fluctuations impacted population stability, connectivity and genetic diversity (Luikart et al. 2019; Gagnaire 2020). These tools provide the opportunity to test whether clownfish species with different degrees of host specialisation responded differently to historical habitat changes.

We hypothesize that Pleistocene sea‐level fluctuations led to repeated coral reef habitat fragmentation, with differential demographic impacts on clownfish species depending on their degree of host specialisation. During glacial periods, lowered sea levels likely reduced reef continuity, constraining gene flow and effective population sizes. We expect generalist species, which associate with multiple anemone hosts, to have maintained more stable population sizes and connectivity across fragmented habitats due to their ecological flexibility. In contrast, specialists, constrained by their narrow host preferences, likely experienced stronger population bottlenecks and greater genetic isolation. These historical processes may have left lasting genomic signatures, with specialists showing reduced genetic diversity, greater population structure and lower connectivity compared to generalists.

We tested these hypotheses by analysing 382 georeferenced genomes from 10 clownfish species exhibiting varying levels of host specialisation. Using Sequential Markovian Coalescent models, we reconstructed the trajectories of historical effective population sizes, identifying bottlenecks and expansions linked to environmental fluctuations. In addition, we used spatial genomic analyses to test the contributions of environmental factors, host specificity and geographic barriers to the structure and connectivity of the present population. Our study provides evidence on the long‐term impacts of habitat fragmentation on Indo‐Pacific reef fish, revealing how historical habitat changes have shaped the evolutionary trajectories and resilience of species under various degrees of specialisation.

## 2 Methods

### 2.1 Sampling and Data Collection

A total of 382 fin clips were collected from 10 clownfish species, representing distinct host categories within the clade classified by Gaboriau et al. (2025). The samples were systematically collected by capturing fish within their host anemones during SCUBA diving expeditions. Samples were preserved either in 70% ethanol (ETOH) or DESS buffer (Oosting et al. 2020) and stored at 6°C immediately after retrieval. Collection efforts spanned multiple locations over an 11‐year period starting in 2012 (detailed information in Extended Data).

### 2.2 Host Association

Species were categorised according to their host preferences following Gaboriau et al. (2025). Two species, *Amphiprion akallopisos* and *A. perideraion* , classified as RM specialists, have evolved to inhabit and reproduce within *Radianthus magnifica*, a solitary sea anemone species with moderate‐length tentacles found in coral reef habitats with moderate water flow. *A. melanopus* and *A. ephippium* , classified as EQ specialists, associate with *Entacmaea quadricolor*, a colonial sea anemone with long tentacles that inhabit crevices and rocky substrates in shallow coral reefs. Finally, *A. sandaracinos* specialised in *Stichodactyla mertensii* and *A. polymnus* specialised in *Stichodactyla haddoni* are classified as SD specialists, as both species belong to the *Stichodactyla* genus, characterised by short‐tentacle solitary anemones forming a carpet‐like extension over sandy areas of coral reefs. The remaining four species examined ( *A. latifasciatus* , *A. akindynos* , *A. chrysopterus* and *A. clarkii* ) were classified as generalists, showing no clear preferences for any specific host sea anemone species.

### 2.3 Environmental and Geographical Data Collection

Sea level dynamics over the last million years were obtained from Müller et al. (2008). Contemporary environmental variables were sourced from the GMED database (Basher et al. 2018) and included oceanographic and biogeochemical parameters such as current velocity, surface current, tide average, temperature, bottom temperature, sea surface temperature range, chlorophyll concentration, phytoplankton concentration, nitrate concentration, salinity, dissolved oxygen range and bottom dissolved oxygen. The environmental data was standardised to a resolution of 0.083° × 0.083°, equivalent to a grid size of $100\;{\mathrm{km}}^{2}$ near the equator and adjusted to the Indo‐Pacific study area. Geographically defined marine regions were obtained from Marine Ecoregions Of the World (MEOW Spalding et al. 2007) and shallow reef habitat locations in the Indo‐Pacific Ocean from UNEP‐WCMC (Unep‐Wcmc and Wri 2010) to implement geographical constraints in species distribution models (SDMs). Oceanic current data and Indonesian Through‐Flow (ITF) polygons were derived from Brown (2007) and Mitsuguchi et al. (2007), respectively, for visualisation and interpretation along with genomic results.

### 2.4 Species Distributions

We estimated distributions of clownfish and their host sea anemones using 3340 occurrences of 28 clownfish species (average 115±150 per species) and 1211 occurrences of the 10 species of sea anemones that host clownfish (average 121±58 per species) sourced from GBIF (GBIF.Org User 2023). Species distribution models were performed using the *ENMTML* R package (de Andrade et al. 2020), using collected environmental predictors to delineate the geographic ranges and distributions of each species. We chose Generalised Linear Models (GLM), Generalised Additive Models (GAM), Maxent with default features (MXD) and Random Forest (RDF) for the modelling process, using a bootstrapping approach for model partitioning, with 5 replicates and 70% of data used for training. Pseudo‐absences were randomly generated in a 1:2 ratio to presences.

Model performance was evaluated using the True Skill Statistic (TSS) and Jaccard index. We applied the Maximum Training Sensitivity plus Specificity (MAX_TSS) and Jaccard thresholds to convert continuous predictions to binary presence‐absence maps. Ensemble forecasting was performed using Principal Component Analysis (PCA) based on TSS values. To account for spatial bias and restrict predictions to ecologically relevant areas, we employed a Minimum Convex Polygon with buffer (MCP‐B) method, using a 500 km buffer around occurrence points. The accessible area for each species was further constrained by using a marine regions shapefile mask. Final species distribution models were used to delineate species ranges, evaluate the spatial representativeness of sampled populations and inform the construction of spatial genetic models (see Spatial Population Connectivity Analysis).

### 2.5 Molecular Data Collection

#### 2.5.1 DNA Extraction, Library Preparation and Sequencing

DNA was extracted using the DNeasy blood and Tissue Kit (Qiagen GmbH, Hilden, Germany) and quantified using the Qubit 2.0 Fluorometer (Thermo Fisher Scientific, Waltham, USA). The integrity of the samples was assessed by electrophoresis. Libraries for samples collected before 2021 were prepared from 100 ng of DNA using the TruSeq Nano DNA library prep standard protocol, while libraries from samples collected after 2021 were prepared using the Nextera DNA Flex Library Preparation Kit following the manufacturer's instructions. Fragment length distribution of the libraries was validated using a Bioanalyzer (Agilent Technologies, Santa Clara, USA). Libraries generated in 2017 and 2018 were sequenced on the Illumina HiSeq 2500 platform with 100 paired‐end lanes, while libraries prepared in 2019 were sequenced on the Illumina 4000 HiSeq platform with 150 paired‐end lanes. Libraries prepared in 2022 and 2023 were sequenced on the NovaSeq 6000 Sequencing System (Illumina, San Diego, California) with 150 paired‐end lanes. Sequencing was performed at the Genomic Technologies Facility (GTF) of the University of Lausanne, Switzerland.

#### 2.5.2 Reads Processing, Mapping and SNP Calling

Low‐quality raw reads were trimmed, and potential adapter contamination was removed using *Trimmomatic* v.0.39 (Bolger et al. 2014) with parameters: ‐‐qual‐ threshold 20 ‐‐length ‐threshold 50. The quality of the processed reads was assessed with *FastQC* v.0.11.9 (Andrews et al. 2012) and *MultiQC* (Ewels et al. 2016). Reads were mapped against the *Amphiprion clarkii* reference genome (GenBank; ID: JALBFV000000000; Moore, Herrera, et al. 2023) using *BWA* v0.7.17 (Li and Durbin 2009), and *Samtools* v1.15.1 (Danecek et al. 2021) was used to process and sort the mapped reads, generating mapping statistics with *Bamtools* v2.5.2 (Barnett et al. 2011). The mapping results were processed to reduce potential SNP calling errors (Altmann et al. 2012). Mapped reads were filtered with *Samtools* v1.15.1 (Danecek et al. 2021) to retain only primary alignments and proper pairs (i.e., at the expected insert size and orientation) with a mapping quality greater than 30. Redundant sequencing data that originated from the overlap of paired reads was removed using the mergeReads task from *ATLAS* v0.9.9 (Link et al. 2017). We used the *ATLAS* pipeline for the SNP call, as it was shown to be more reliable than *GATK* in non‐model species, maintaining high accuracy in variant calling for moderately divergent species (Duchen and Salamin 2021). Genotype likelihoods (GL) were calculated at each position using the GLF task from *ATLAS* for each sample and the reference genome. The genotype likelihoods were then used to infer the major and minor alleles for each position running the majorMinor task, obtaining the genotype of the individuals. The SNP calling pipeline was carried out with all individuals from all species together, generating an all‐species dataset, and for each species independently, using all individuals from multiple populations, generating species‐specific datasets.

#### 2.5.3 Variant Filtering and Data Processing

Reliability of the SNP dataset was improved by filtering invariant or poor confidence positions with *vcftools* v1.15.1 (Danecek et al. 2021). We filtered out low‐confidence positions (variant quality score < 30), positions with coverage higher than 50, and with more than 10% of missing data (parameters: ‐‐minQ 30‐‐max‐missing 0.9‐‐minDP 5‐‐max‐meanDP 30‐‐maxDP 50). The VCF files were divided by species populations to estimate individuals' heterozygosities (‐‐het) and minor allele frequencies (‐‐maf) using *vcftools* v1.15.1 (Danecek et al. 2021). With this information, we removed rare variants suspected to be private alleles or sequencing errors using a MAF threshold of 0.02. In addition, we merged species population VCF files for each species, keeping only common variants using merge task from *bcftools* v1.15.1 (Danecek et al. 2021). Finally, VCF files were LD pruned and converted to BED files using *Plink* v.1.90 (Purcell et al. 2007). The resulting dataset was used for all subsequent genomic analyses except demographic history reconstructions.

### 2.6 Genetic Diversity and Population Structure

We evaluated species population structure using Admixture (Alexander et al. 2009) and Principal Component Analysis (PCA) with *Plink* v.1.9 (Purcell et al. 2007), in order to identify genetic clustering patterns between and within species. We carried out Admixture and PCA on each species BED file to determine population identity and assess population structure, crucial for interpretations of the Sequential Markov Coalescence model (Mather et al. 2020). Admixture was run with *K* from 1 to the number of assumed populations of each species plus three, with 10‐fold cross‐validation and 2000 bootstraps. Following Bradburd et al. (2018), the best *K* value was selected on the lowest cross‐validation error. We also carried out PCA on all‐species data sets to determine species identities. Three *A. polymnus* samples were clustering with *A. clarkii* showing to be potentially misidentified or mislabelled. Consequently, these three samples were removed for downstream analysis.

Nucleotide diversity (π) was calculated as a measure of within‐population genetic diversity using filtered VCF files for each species population. Nucleotide diversity (π) was calculated per site for each population using ‐‐site‐pi with *vcftools* v1.15.1 (Danecek et al. 2021). π values were averaged over 10 kb windows and include only populations with sample sizes greater than one. Between‐population differentiation was assessed estimating ($F_{\mathrm{ST}}$) using the ‐‐weir‐fst‐pop function in *vcftools* and the absolute divergence between populations ($d_{\mathrm{xy}}$) across non‐overlapping 50 kb windows using popgenWindows.py from *genomics_general* repository (https://github.com/simonhmartin/genomics_general). All three genome‐wide metrics were averaged and summarised either at the population level (π) or at the pairwise population level ($F_{\mathrm{ST}}$ and $d_{\mathrm{xy}}$).

To enable cross‐species comparisons, we accounted for potential confounding effects of geographic distance and population split time (see Reconstruction of Demographic Histories section for details on split time inference). We applied a posterior adjustment to the original $F_{\mathrm{ST}}$ and $d_{\mathrm{xy}}$ values to account for the influence of divergence time and geographic distance. Specifically, we fitted linear mixed models in which $F_{\mathrm{ST}}$ and $d_{\mathrm{xy}}$ were modelled separately as a function of geographic distance, population divergence time and host category. To properly account for non‐independence in pairwise comparisons—where the same population can be involved in multiple comparisons and may be shared across species—we included crossed random intercepts for each population (Pop1 and Pop2), as well as a random intercept for species. This structure allowed us to control for both species‐level effects and repeated use of populations across comparisons. To obtain adjusted estimates of genetic differentiation that control for geographic and temporal effects, we then removed the contributions of divergence time and geographic distance fixed effects from the original $F_{\mathrm{ST}}$ and $d_{\mathrm{xy}}$ values. This approach preserves species‐ and population‐level variation captured by the random effects while focusing on differentiation independent of distance and divergence time.

Results were visualised using *R* v.4.3.1 (R Core Team 2021), with custom scripts for population‐specific summaries. These metrics were used to characterise genetic diversity and differentiation, supporting interpretations of ecological and historical processes shaping population dynamics.

### 2.7 Reconstruction of Demographic Histories

We employed *msmc2* (Schiffels and Wang 2020) to reconstruct the historical dynamics of effective population size ($N_{e}$) across all populations of the 10 clownfish species, following guidelines from *msmc‐tools* (https://github.com/stschiff/msmc‐tools). Populations were defined based on broad geographic sampling regions (e.g., Thailand, Indonesia). These definitions were then validated using ADMIXTURE and PCA, which confirmed the absence of within‐population structure (see previous section and Figure S6). This validation supported the use of each geographic region as a distinct population unit for MSMC2 analyses.

Genomic data processing for demographic reconstructions followed a separate pipeline from the one described in the previous section. Specifically, from individual BAM files, we used mpileup and call tasks in *bcftools* v1.15.1 (Danecek et al. 2021) followed by bamCaller.py from *msmc‐tools* to generate individual VCF files at the chromosome level. Average coverage by chromosome was estimated using the depth task in *Samtools* v1.15.1 (Danecek et al. 2021). VCF files were phased with *Whatshap* (Martin et al. 2016), and individual consensus masks and masks were generated using makeMappabilitymask.py. Then, *msmc2* input files were generated with generate_multihetsep.py.

For each species and population, we performed 10 independent demographic reconstructions, each based on a random subset of three individuals from the population, as an additional measure to account for potential cryptic within‐population structure. The time‐segmented pattern used was 1×2+16×1+1×2, recommended for shorter genomes (Schiffels and Wang 2020), with a maximum of 100 iterations. Furthermore, we performed 50 bootstraps using multihetsep_bootstrap.py to assess uncertainty levels at each time step. Historical time periods and $N_{e}$ were inferred using a fixed mutation rate per generation of $4\times 10^{-8}$ (Delrieu‐Trottin et al. 2017; Rolland et al. 2018) and a generation time of 5 years (Buston and García 2007), consistent with previous clownfish studies (Schmid, Bachmann Salvy, et al. 2024; Marcionetti et al. 2024).

We processed the *msmc2* output data using R v.4.3.2 (R Core Team 2021). To ensure the reliability of the estimates, we first removed the identified bootstraps from *A. sandaracinos* showing unrealistic and abnormally high $N_{e}$ values at mid‐time points. Specifically, bootstraps from the Australian population of *A. sandaracinos* were completely removed, while 9 out of 10 individual‐based bootstraps from the Papua New Guinea population were excluded. We identified these bootstraps as outliers by selecting those with $N_{e}$ values that exceeded a threshold based on the 97.5% percentile of $N_{e}$ values across all bootstraps for that species and period of time. This filtering approach aimed to exclude bootstraps with anomalously high $N_{e}$ estimates, which may arise from random sampling of individuals that exhibit signs of hybridization among species (He et al. 2019; Schmid, Hartasanchez, et al. 2024), or individual combinations that contain migrants that may accentuate population substructure within the bootstrap input data (Novo et al. 2023). Furthermore, we filtered out unrealistic $N_{e}$ estimates at the time edges applying a threshold defined as the 95% quantile of $N_{e}$ values at all time points, removing values above this threshold to ensure robust population reconstructions, as these often represent periods with limited coalescent information (Li and Durbin 2011).

Furthermore, we inferred pairwise population split times from *msmc2* demographic reconstructions, using plot_utils.py and combineCrossCoal.py from the *msmc‐tools* repository. This process involved comparing the cross‐coalescence rate between populations to their within‐population coalescence rates. The split time is estimated as the point where the cross‐coalescence rate drops to half of the within‐population rate.

### 2.8 Demographic Responses to Host Specialisation and Pleistocene Sea‐Level Fluctuations

Population‐level $N_{e}$ reconstructions for each species—including bootstraps—were used to test the effects of host preference and sea‐level fluctuations. Species‐level averages were computed for simplified visualisation of species overall demographic trends. Historical $N_{e}$ values were calculated using the weighted harmonic mean, which adjusts for the relative time intervals to account for the unevenness of the data set. To standardise the time scale across all reconstructions, we calculated the midpoint between the left‐ and right‐time boundaries for each estimate. We then created a common time grid using 100 logarithmically spaced points between 1 and the maximum time point (rounded to the nearest million years). $N_{e}$ values were interpolated onto this common time grid for each species, population and bootstrap combination using linear interpolation. Time points outside the time range of the demographic reconstruction were kept as NA. Historical sea level data from Müller et al. (2008) were incorporated into our analysis. The sea level data were aligned with our time grid, focusing on the period relevant to our demographic reconstructions.

We used a Generalised Linear Mixed Model (GLMM) with the *MASS* R package (Venables and Ripley 2002) to investigate the effects of sea level, ecological specialisation, geographical location and time on $N_{e}$ The model included $N_{e}$ as the response variable, with fixed effects for scaled sea level, host category, geographic location (population) and scaled time, including all their two‐ and three‐way interactions. Random effects accounted for hierarchical structure, with species, population nested within species, and bootstrap replicates nested within population. The full model formula was:

$$\begin{array}{c} N_{e}∼\text{scale}\;\left(\text{seaLevel}\right)\times \text{hostCategory}\times \text{scale}\;\left(\text{time}\right)\\ +\mathrm{pop}+\left(1|\text{species}/\mathrm{pop}/\text{bootstrap}\right) \end{array}$$

fitted with a Gamma distribution and a log link to appropriately model the strictly positive and continuous nature of the response variable, providing a robust model for assessing the effects of these factors on effective population size.

We performed Functional Data Analysis (FDA) to compare $N_{e}$ trajectories between generalist and specialist species using the *fdANOVA* R package (Górecki and Smaga 2019). This approach allowed us to analyse the entire $N_{e}$ trajectory as a functional response, providing information on the overall temporal patterns of demographic change between ecological types.

### 2.9 Spatial Population Connectivity Analyses

To understand the spatial genetic structure of populations, we applied Estimated Effective Migration Surfaces (*EEMS*) (Petkova et al. 2016) and Migration and Population‐size Surfaces (*MAPS*) (Al‐Asadi et al. 2019). *EEMS* infers relative rates of effective migration under equilibrium, providing a time‐averaged view of population structure, while *MAPS* estimates absolute migration rates and population sizes using long pairwise shared coalescence segments, revealing temporal variations in dispersal rates and population sizes.

*EEMS* estimates effective migration rates and genetic diversity patterns within and between populations using genetic data, modelling spatial gene flow patterns. Using Markov Chain Monte Carlo (MCMC) simulations, *EEMS* infers these rates between the demes, providing information on genetic structure, barriers to gene flow and dispersal dynamics. It also calculates a posterior probability for each estimate, offering a probabilistic assessment of whether rates are higher or lower than expected under the Isolation‐by‐Distance assumption. This Bayesian approach facilitates a nuanced understanding of genetic connectivity and the impact of spatial factors on population dynamics and evolutionary processes.

Genetic dissimilarities between pairs were calculated using the bed2diffs program, available within the *EEMS* toolkit. All analyses were executed with 2,000,000 iterations following a burn‐in phase of 1,000,000 iterations for the Markov Chain Monte Carlo. For each species, we performed analyses with 50, 200 and 500 demes, employing three chains for each configuration. This extensive exploration allowed us to compare results and fine‐tune parameters, following the developers' recommendations. The results of these three runs were visualised using the *rEEMSplots* R package (Petkova et al. 2016), demonstrating consistent results across runs. Species *A. latifasciatus* and *A. ephippium* were excluded from these analyses due to having only one population of each.

*MAPS* was employed to spatially estimate the dispersal rates and population densities of the 10 clownfish species. This method utilises a coalescence‐based Bayesian approach to infer migration rates and population sizes from genetic similarity matrices derived from Identity‐By‐Descent (IBD) segments of predetermined lengths. Using Markov Chain Monte Carlo (MCMC) processes, *MAPS* detects patterns at specific time periods, thereby providing a nuanced understanding of genetic connectivity, population dynamics and the impacts of spatial and temporal factors on evolutionary processes.

Genetic data for each clownfish species were processed using the *MAPS* toolkit (https://github.com/halasadi/ibd_data_pipeline), which provides a comprehensive pipeline for the inference of the IBD segment. We used BED files as input and employed the toolkit's Snakemake‐based workflow to infer Identity‐By‐Descent (IBD) segments using *Beagle* v.4.1 (Browning and Browning 2011). Estimated *A. clarkii* recombination maps (see next section) were used to obtain recombination distances and identify IBD segments. We targeted segments spanning five length ranges: 6‐Inf cM, 2–6 cM and 0.02–1 cM. These ranges correspond approximately to time periods of 62.5, 187.5, 250, 3750 and 19,125 years ago, respectively. This correspondence between IBD segment length and time periods was computed using the equation provided in the Supporting Information of Al‐Asadi et al. (2019). We conducted test runs using recombination maps from multiple *A. clarkii* populations, including both subsets from Indonesia and Papua New Guinea. These tests yielded comparable results on all maps and time periods. Based on these findings, we selected the *A. clarkii* Indonesia subset A recombination map for our subsequent analyses, as it provided a representative model for the recombination landscape of the species.

To ensure robust and reliable results, we performed a comprehensive analysis using *MAPS* for each clownfish species. We used three different deme configurations (50, 200 and 500). For each configuration, we ran three independent Markov Chain Monte Carlo (MCMC) chains, enhancing our ability to assess convergence and stability of the results. Each MCMC chain was run for 2,000,000 iterations, with the first 1,000,000 iterations discarded as burn‐in to allow the chain to reach equilibrium. We sampled parameter values every 1000 iterations, resulting in 1000 samples per chain for posterior inference. This extensive exploration allowed us to compare results across different parametrization and assess the consistency of our findings. We used this approach to fine‐tune parameters and ensure the robustness of our estimates. The outcomes of these runs were visualised using the *MAPS* plotting functions, available at https://github.com/halasadi/plotmaps.

### 2.10 *Amphiprion clarkii* Recombination Map

We estimated the recombination maps of *A. clarkii* populations in Indonesia and Papua New Guinea, employing pyrho v.0.7.0 (Spence and Song 2019) aiming at fine‐scale recombination rates across the genome based on linkage disequilibrium patterns. pyrho was selected for its ability to account for demographic history (i.e., temporal changes in $N_{e}$) and its robust performance with phased and unphased data (Spence and Song 2019). However, each population was randomly divided into two subsets—A and B—with 24 individuals each for Papua New Guinea and 19 and 18, respectively, for Indonesia, to assess the consistency of recombination rate estimates and validate the robustness of our methodology.

The recombination rate inference process consisted of two main steps. First, we constructed likelihood tables for biallelic sites using the make_table command. This step incorporated a mutation rate (μ) of $4\times 10^{-8}$, population‐specific demographic histories inferred by SMC++ v.1.15.4 (Terhorst et al. 2017), and the Moran approximation (‐‐approx flag). We set the ‐‐moran_pop_size parameter to 1.5 times the number of haplotypes in each population, as recommended by pyrho developers.

We used a fixed window size of 50 and performed simulations mimicking under different evolutionary characteristics (μ, sample size, $N_{e}$), to optimise pyrho block penalty. Through this process, we identified an optimal block penalty of 15, which minimised both false positives and false negatives, and applied it in the optimise command to generate the final recombination map. The resulting pyrho output provided estimates of the per base, per generation recombination rate (r) between adjacent SNP pairs. To ensure compatibility with IBD segment inference in the *MAPS* workflow, we converted the recombination rates (r) to centimorgans per megabase (cM/Mb) and reformatted the recombination maps accordingly to meet the required input format.

## 3 Results

**FIGURE 1:**
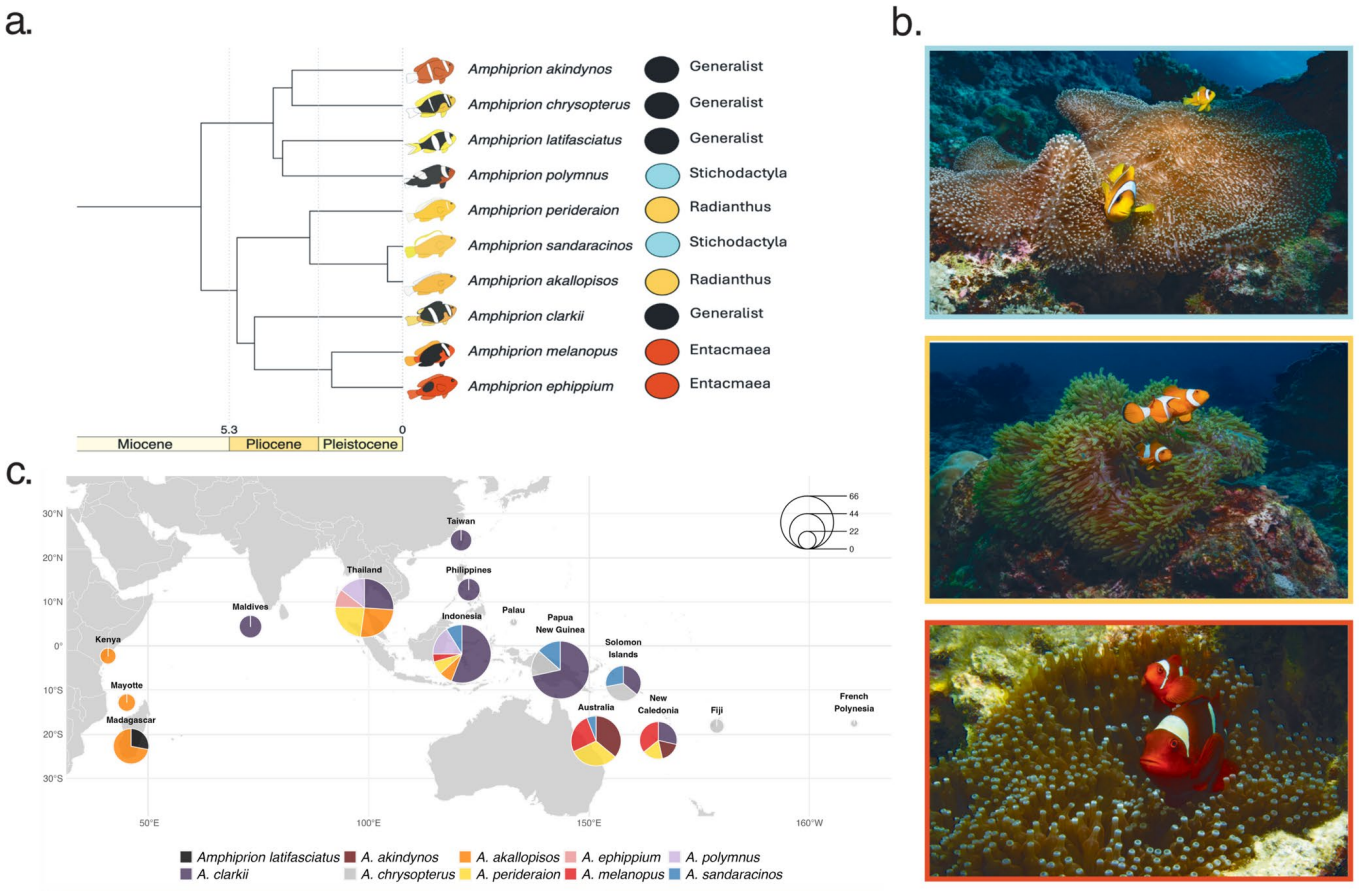
Species in this study. (a) Subset of phylogenetic relationships from Gaboriau et al. (2025), showing studied species and host specialisation types (coloured circles). (b) Sea anemone exemplars for each host specialisation: *Stichodactyla mertensii* (light blue), *Radianthus magnifica* (orange) and *Entacmaea quadricolor* (red). Credit: Lucy Fitzgerald. (c) Sample distribution by species. Pie charts illustrate the proportional representation of species at each location, with the corresponding country name indicated above each chart and chart size reflecting the total number of samples collected in the region (see legend at top right). Colours denote different species, as indicated below.

We generated whole genome sequencing data from 382 georeferenced individuals to analyse the genetic structure and reconstruct the demographic histories of multiple populations in 10 clownfish species with varying host specialisations (Figure 1). Sequencing reads were mapped onto the *Amphiprion clarkii* reference genome (Moore, Herrera, et al. 2023), with mapping rates ranging from 81.05% to 97.74%. Average sequencing depths varied between 5.82X and 42.21X and species‐specific SNP datasets contained between 202,007 and 2,192,819 SNPs (detailed information in Extended Data).

### 3.1 Demographic Histories Reveal Differential Impacts of Pleistocene Sea Level Fluctuations

**FIGURE 2:**
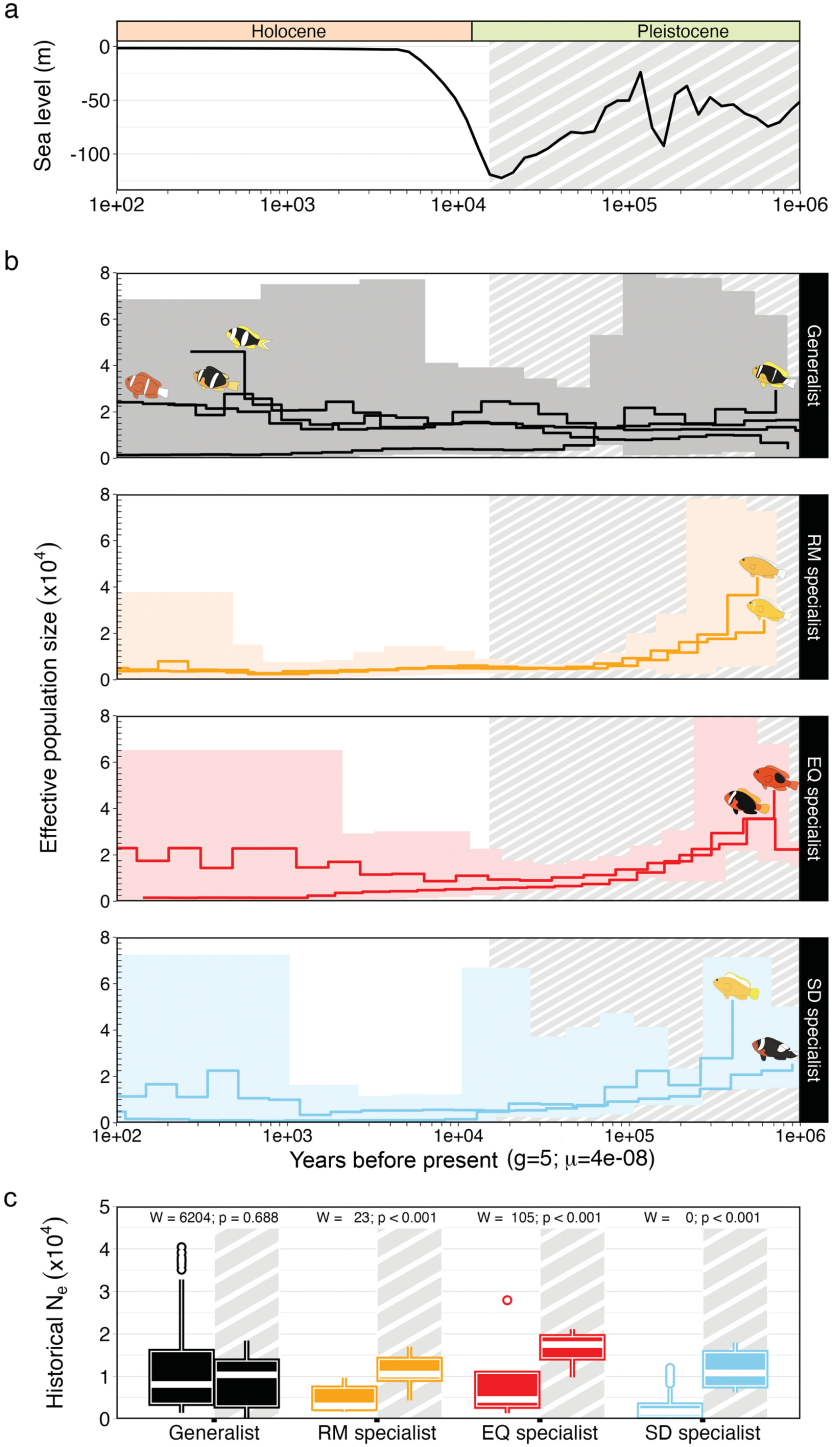
Demographic dynamics of clownfish species in relation to late Pleistocene sea level changes. (a) Sea level fluctuations over the past 1 million years. Grey striped areas indicate periods of increased sea level fluctuations with a declining trend. Geological epochs are indicated at the top. The log scaled *x*‐axis represents years ago; *y*‐axis indicates sea level in meters relative to present levels. Data sourced from Müller et al. (2008). (b) Effective population size ($N_{e}$) reconstructions using *msmc2* for ten clownfish species, grouped by host association: generalists (black), *Radianthus* specialists (orange), *Entacmaea* specialists (red) and *Stichodactyla* specialists (blue). Solid lines show species averages; colour‐shaded areas represent the range across populations. Species illustrations are placed near the highest $N_{e}$ values. Grey striped areas correspond to periods of sea level fluctuations. (c) Comparison of historical $N_{e}$ (calculated as weighted harmonic means) between periods of sea level fluctuations (striped background) and subsequent rise, across host specialisation groups. Boxes show interquartile range with median (white line); whiskers indicate upper and lower bounds, and white points mark outliers. Kruskal–Wallis test results are shown above each plot.

We investigated historical changes in effective population size ($N_{e}$) of clownfish species using *msmc2* (Schiffels and Wang 2020) (Multiple Sequentially Markovian Coalescence) on whole‐genome SNP data (Figure S1). We found contrasting demographic trajectories between generalist and specialist clownfishes during the Quaternary, particularly in response to late Pleistocene sea‐level fluctuations (Figure 2).

Generalist species maintained relatively stable $N_{e}$ with greater variability between populations. In contrast, all three specialist groups (*Radianthus*, *Entacmaea* and *Stichodactyla* specialists) exhibited significant and sustained $N_{e}$ declines across populations beginning around 300,000 years ago, coinciding with major sea‐level regressions during the late Pleistocene. These declines persisted across subsequent glacial cycles (notably 350–150 kya and 120–15 kya), suggesting long‐term demographic impacts potentially linked to habitat loss and fragmentation driven by repeated sea‐level lowstands. Functional Analysis of Variance (FANOVA) confirmed the statistical significance of the differences between ecological groups (F=4,477.334, p < 0.001).

During the early late Pleistocene (>500,000 years ago), specialist species exhibited significantly higher historical $N_{e}$ than generalists ($\chi^{2}=28.308$, df=1, p < 0.001). However, prolonged and drastic $N_{e}$ declines in specialist populations resulted in recent $N_{e}$ estimates (< 15,000 years ago, Holocene) significantly lower than their late Pleistocene levels (*Radianthus* specialists: W=23, p < 0.001; *Entacmaea* specialists: *W* = 105, *p* < 0.001; and *Stichodactyla* specialists: *W* = 0, *p* < 0.001) and below contemporary estimates of generalists ($\chi^{2}=38.506$, df=1, p < 0.001). In contrast, generalist species maintained relatively stable $N_{e}$ across periods of sea level change (W=6,163, p=0.638), with most showing an upward trend starting around 100,000 years ago, except for *A. chrysopterus* .

During the Holocene, specialist populations exhibited persistently low $N_{e}$, failing to show significant recovery despite the rise in sea level. These $N_{e}$ values not only remained lower than pre‐late Pleistocene levels but also reached extremely low levels in some populations within the last 1000 years. The most extreme case was observed in the Papua New Guinea population of *A. sandaracinos* , where the average $N_{e}$ dropped to only 100 individuals contributing to the next generation. In contrast, the Indonesian population of *Entacmaea* specialist *A. melanopus* stood out among specialists, reaching a high $N_{e}$ of 65,275±5.42 individuals. In generalists, Pacific populations of *A. chrysopterus* and *A. clarkii* showed declining trends similar to those of specialists, indicating local effects on $N_{e}$ dynamics (Figure S2).

A generalised linear mixed model (GLMM) was used to assess the influence of time, sea level, geographical location and host preference on $N_{e}$, considering species and bootstrap replicates used to estimate variation of $N_{e}$ (see Material and Methods) as random factors. According to the GLMM, sea level fluctuations had a small but significant negative effect on $N_{e}$ (β=−0.036, p < 0.050). Host category significantly influenced $N_{e}$, with *Radianthus* and *Stichodactyla* specialists showing significant negative effects compared to generalists (β=−0.348 and β=−0.321, respectively; p < 0.050). The interaction between sea level, host preference and time was highly significant for all specialist groups (p < 0.001), indicating complex relationships in determining $N_{e}$ dynamics (Table S1).

### 3.2 Connectivity Patterns Driven by Ecological Specialisation and Dispersal Dynamics

We investigated the spatial genetic structure of clownfish populations using Estimated Effective Migration Surfaces (*EEMS*; Petkova et al. 2016) and Migration And Population‐size Surfaces (*MAPS*; Al‐Asadi et al. 2019). These complementary approaches provided insight into both time‐averaged population structure and temporal variations, from the Last Glacial Maximum (LGM) between 26,500 and 19,000 years ago to present‐day dispersal rates and population sizes.

**FIGURE 3:**
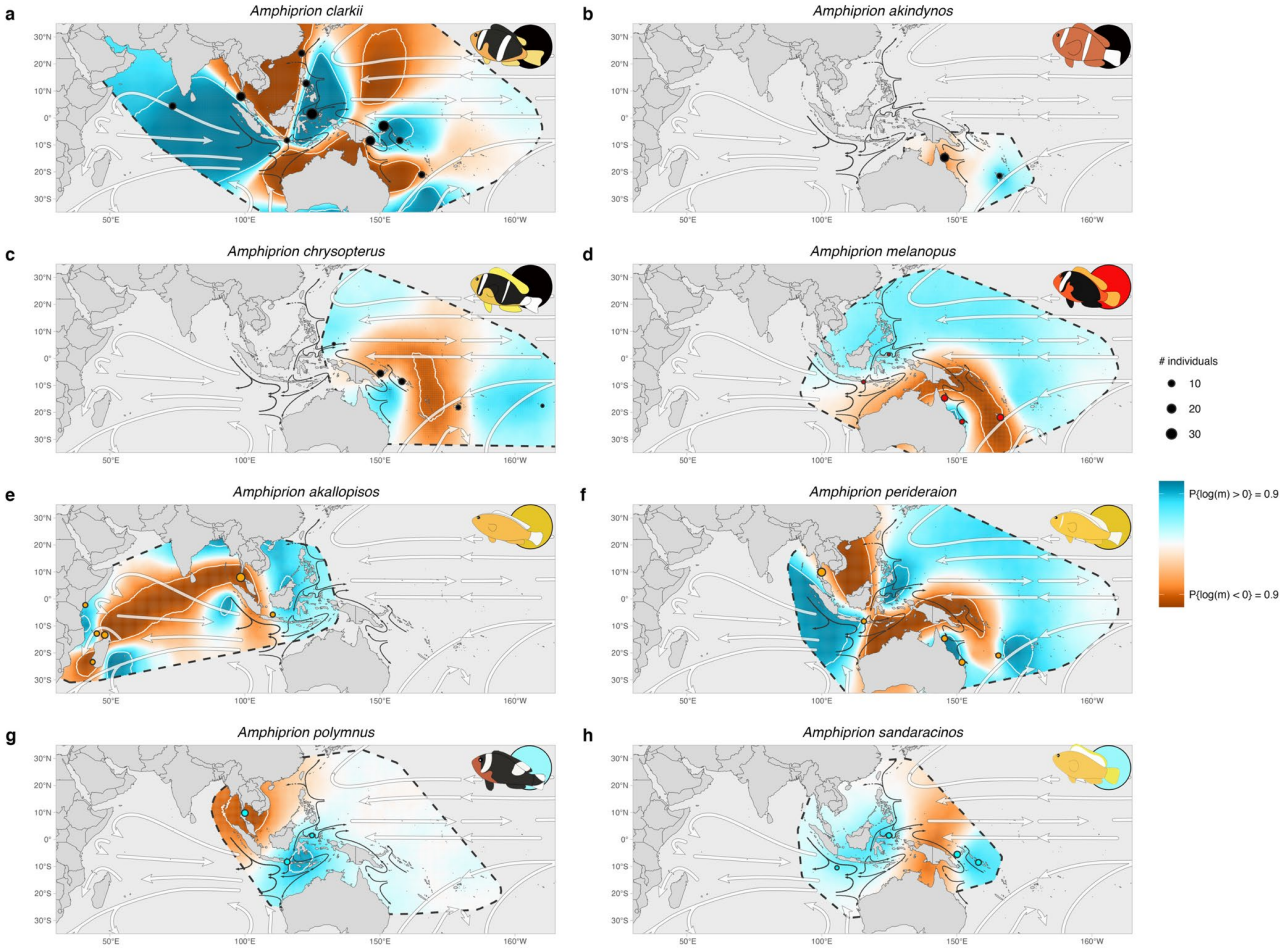
Spatial patterns of population connectivity. Maps show the clownfish clade distribution range. The dashed polygon indicates the area used for inference, based on a 1000 km buffer around the species distributions. Sampling locations are indicated by circles, with size representing the number of individuals, and colour the host category (black‐generalists, orange‐*Radianthus*, red‐*Entacmaea*, blue‐*Stichodactyla*). White arrows indicate global oceanic currents obtained from Browning and Browning (2011), while dark arrows indicate Coral Triangle currents sourced from Mitsuguchi et al. (2007). Colour shade indicates the posterior probability of a location having higher (blue) or lower (red) migration than expected under Isolation‐by‐distance (IBD). Species names are shown at the top, and vector illustrations and coloured circle indicating the host category on the top‐right corner.

Generalist species displayed larger areas of higher migration rates than expected under isolation‐by‐distance based on *EEMS* analyses, often found connecting populations across geographic ranges ($P\left(\log\left(m\right)>0\right)>0.9$; Figure 3). For instance, *A. clarkii* showed the highest effective migration rates, nearly double those predicted by an isolation‐by‐distance model, in regions linking multiple populations within the Coral Triangle, which includes Indonesia, Malaysia, the Philippines, Papua New Guinea and northern Australia. In contrast, specialist species showed predominantly areas of reduced effective migration rates ($P\left(\log\left(m\right)<0\right)>0.9$), with values 30–80 times lower than expected under isolation‐by‐distance. Areas of low migration in specialists were often located between populations, indicating geographical barriers to gene flow that may contribute to reproductive isolation.

Despite varying geographic distributions and ecological specialisations, we were able to identify consistent dispersal corridors and geographical barriers between species. The Coral Triangle showed migration rates for *A. clarkii* , *A. perideraion* and *A. polymnus* that were surprisingly high, demonstrating its role as a significant dispersal pathway for both specialist and generalist species. High migration rates were also associated with major local currents, such as the Indonesian Through‐Flow, highlighting the role of marine currents in facilitating connectivity between populations (Figure 3). In contrast, shallow continental shelves (Sunda and Sahul Shelves), the Melanesia archipelago and vast oceanic regions consistently hindered gene flow between populations, exhibiting low effective migration rates.

**FIGURE 4:**
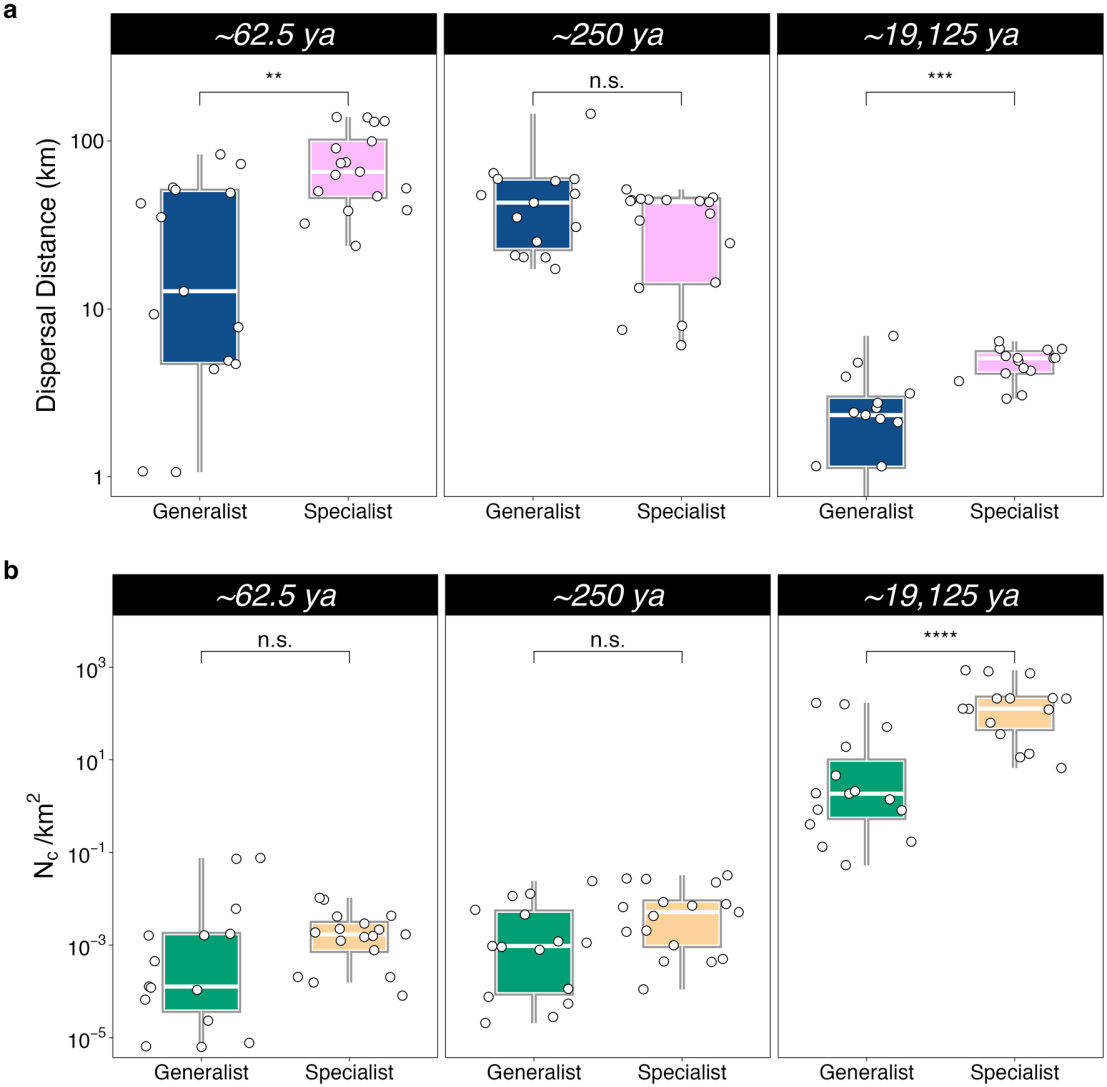
Temporal patterns of dispersal distance and population density across ecological strategies (generalist vs. specialist). (a) Dispersal distance (km) and (b) population density ($N/km^{2}$) were calculated per species and population by averaging values across all locations within the marine ecoregion that encompasses each population, as defined by the Marine Ecoregions of the World (MEOW) classification. These estimates were derived from *MAPS* analyses across three time points (∼62,500, ∼25,000 and ∼19,125 years ago). Results are grouped by behavioural strategy to facilitate comparisons between generalist and specialist species. Boxplots display the interquartile range, with the central line representing the median and whiskers extending to 1.5 times the interquartile range. Statistical significance between generalists and specialists at each time point is indicated as follows: n.s. (not significant), * (p<0.05), ** (p<0.01), *** (p<0.001), and **** (p<0.0001).

*MAPS* temporal analysis revealed increased dispersal from the LGM to present (Figure 4a, Figure S3). Specialists exhibited significantly higher dispersal distances than generalists during the LGM (specialists: 4.47±1.05 km; generalists: 2.55±1.90 km; W=38, df=1, *p* < 0.001). This difference persisted after the increase in dispersal distances during sea level rise (specialists: 76.8±42.0 km; generalists: 38.0±23.5 km; W=52, df=1, *p* < 0.01). The inferred spatial patterns of dispersal over time largely corresponded to time‐averaged migration patterns identified by *EEMS* analyses, suggesting congruence in the detection of dispersal corridors and geographical barriers (Figure S4). These results indicate that the identified geographical barriers and dispersal corridors are well‐established within the biogeography of clownfish and were likely established long before the LGM, shaping long‐term population connectivity patterns.

### 3.3 Contemporary Population Structure Reflects Historical Dynamics

Analyses of population structure and genetic diversity revealed different patterns between generalist and specialist clownfish species, reflecting their historical dynamics and ecological adaptations (Figure S5). Admixture analyses and Principal Component Analysis (PCA) indicated limited genetic structure within species, with specialists exhibiting a higher genetic structure relative to their geographically defined populations than generalists (Figure S6).

**FIGURE 5:**
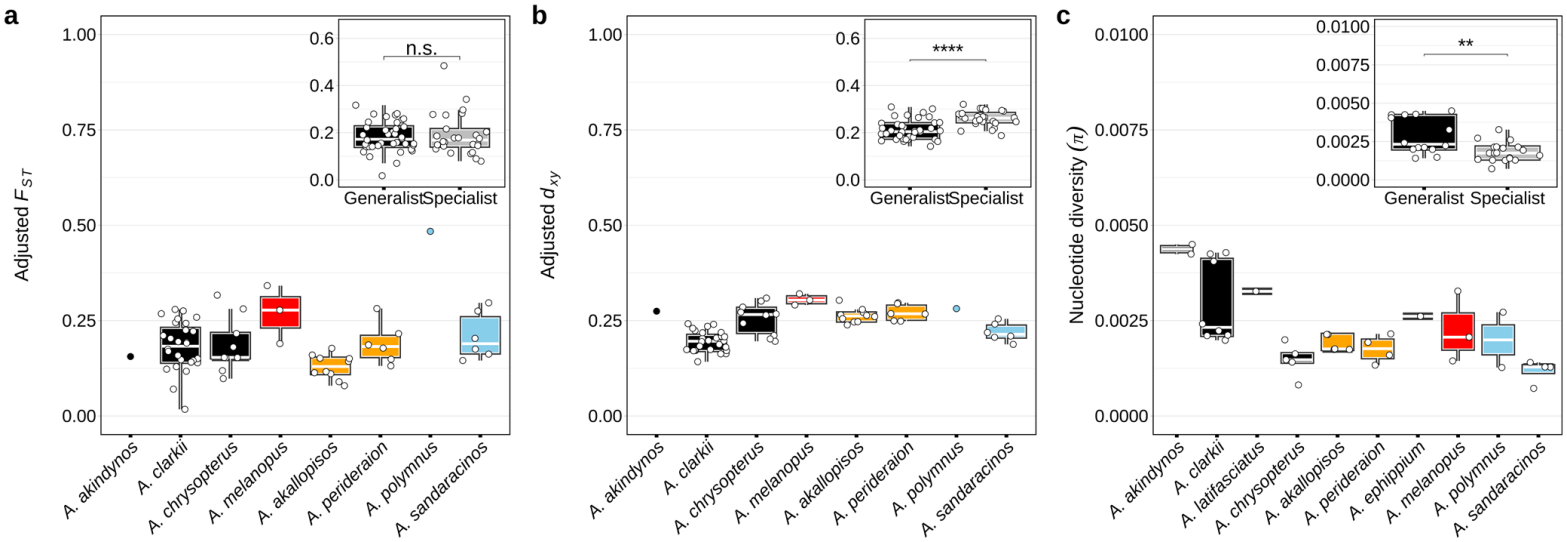
Genetic structure and diversity across clownfish species. (a) Adjusted $F_{\mathrm{ST}}$ values were calculated by removing the effects of geographic distance and divergence time from the original $F_{\mathrm{ST}}$ values. A linear mixed model was used, where $F_{\mathrm{ST}}$ was modelled as a function of geographic distance, divergence time and behaviour, with random intercepts for species. (b) $d_{\mathrm{xy}}$ values reflecting pairwise sequence divergence between populations. $d_{\mathrm{xy}}$ values were adjusted by removing the effects of geographic distance and divergence time following a similar procedure as for Adjusted $F_{\mathrm{ST}}$. (c) Nucleotide diversity (π) reflects within‐population genetic diversity for each species. Only populations with more than one sample are included. Boxplots display the distribution of values, with the box indicating the interquartile range, the central line representing the median, and whiskers extending to 1.5 times the interquartile range. Boxplots are coloured according to host preferences: generalists (black), *Radianthus* specialists (orange), *Entacmaea* specialists (red) and *Stichodactyla* specialists (light blue). Values are averaged over 10 kb windows along chromosomes for each species. Inset boxplots in each panel compare adjusted $F_{\mathrm{ST}}$, adjusted $d_{\mathrm{xy}}$ and π values between generalists and specialists with Kruskal–Wallis test results reported as follows: ** (p<0.01), *** (p<0.001), and **** (p<0.0001).

Linear mixed model (LMM) analyses of pairwise population $F_{\mathrm{ST}}$ values revealed significant effects of host specialist/generalist behaviour. While divergence time (β=0.07, 95% CI = [0.02, 0.13], p=0.005) and geographical distance (β=0.05, CI = [0.00, 0.10], p=0.049) were a significant positive predictor of $F_{\mathrm{ST}}$, host specialisation showed a marginal but non‐significant effect (β=0.18, CI = [−0.01, 0.35], p=0.052; Figure 5a). However, interaction effects between geographical distance and behaviour (β=−0.27, CI = [−0.44, −0.10], p=0.003) and the three‐way interaction with divergence time (β=−0.26, CI = [−0.44, −0.07], p=0.007) were both significant, indicating that the effects of spatial and temporal distance on genetic differentiation differ by behaviour (Table S2a).

Similarly, LMM results for absolute genetic divergence ($d_{\mathrm{xy}}$) identified divergence time (β=0.04, CI = [0.02, 0.06], p<0.001) and geographical distance (β=0.03, CI = [0.01, 0.05], p=0.004) as significant predictors, with host specialisation showing a marginal effect (β=0.08, CI = [−0.00, 0.15], p=0.053). A significant interaction between divergence time and behaviour was detected (β=0.06, CI = [0.01, 0.12], p=0.022), suggesting that specialists accumulate genetic differences more rapidly over time than generalists (Table S2b).

When examining absolute genetic divergence independent of the fixed effects of spatial and temporal distance (i.e., adjusted $d_{\mathrm{xy}}$), specialists exhibited significantly higher genetic divergence compared to generalists (Figure 5b). This adjusted analysis isolates the effect of host specialisation on genetic differentiation by accounting for confounding spatial and temporal factors, thereby revealing a clearer and more direct association between specialisation and genetic divergence than the full LMM alone. Note that this adjusted approach removes confounding spatial and temporal effects, clarifying the impact of host specialisation, but it overlooks important interactions present in the full model.

In terms of genetic diversity, generalist species showed a higher median nucleotide diversity (π) than specialists, except for *A. chrysopterus* , which had π values comparable to those of specialists. However, the overall difference in genetic diversity between generalists and specialists was statistically significant ($\chi^{2}=11.724$, df=1, p < 0.01; Figure 5c).

**FIGURE 6:**
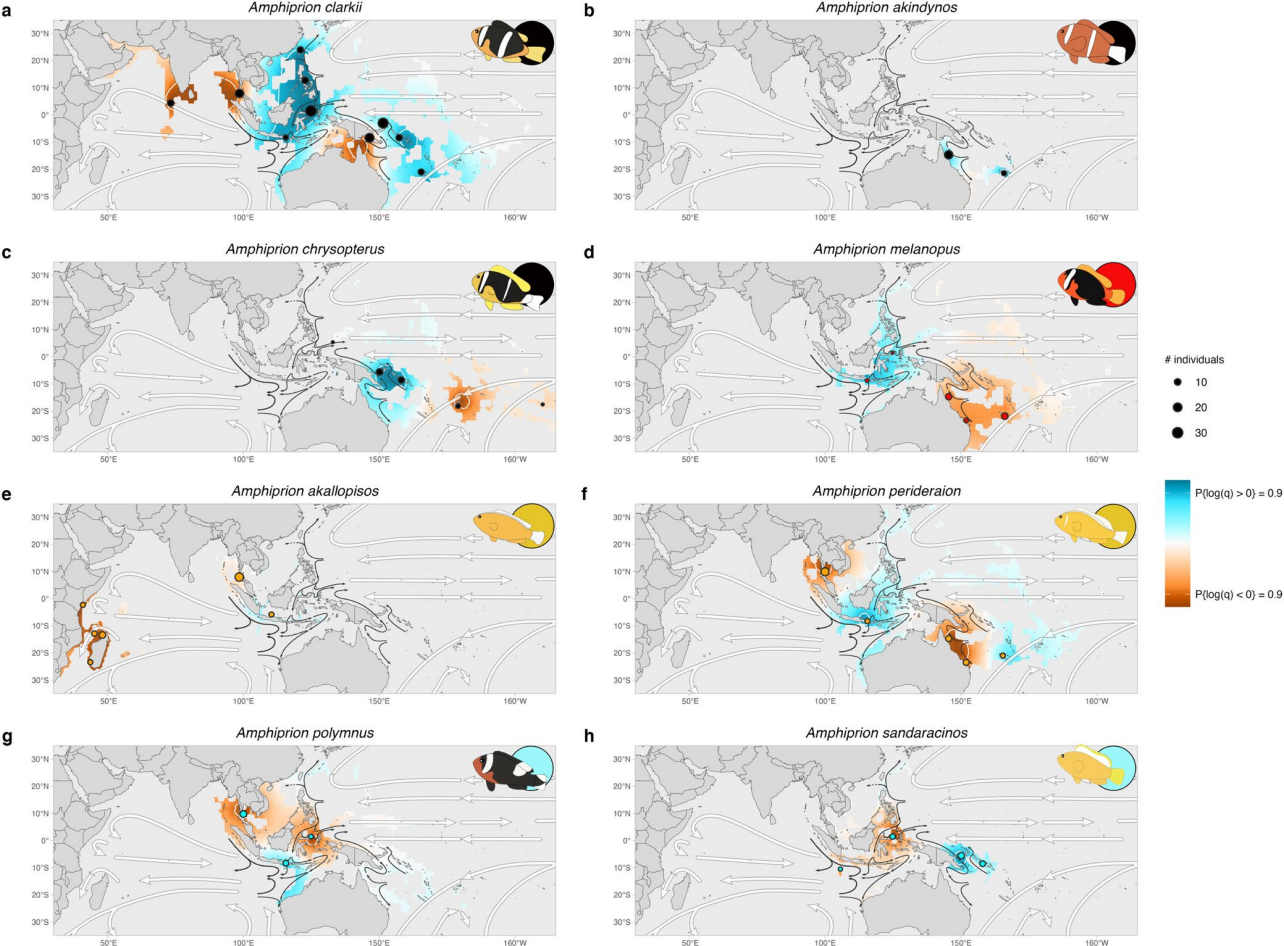
Spatial patterns of genetic diversity. Maps show the clownfish clade distribution range. Sampling locations are indicated by circles, with size representing the number of individuals and colour the host category (black‐generalists, orange‐*Radianthus*, red‐*Entacmaea*, blue‐*Stichodactyla*). Effective genetic diversity surfaces are masked by inferred species distributions. White arrows indicate global oceanic currents, while dark arrows indicate Coral Triangle currents. Colour shade indicates the posterior probability of a location having higher (blue) or lower (red) genetic differentiation than expected under Isolation‐by‐distance. Species names are shown at the top, and vector illustrations and coloured circle indicating the host category on the top‐right corner.

*EEMS* analysis revealed distinct spatial patterns of within‐population genetic diversity between generalist and specialist clownfish species. Widely distributed generalists, *A. clarkii* and *A. chrysopterus* , exhibited significantly higher genetic diversity in core regions than expected under the isolation‐by‐distance model ($P\left(\log\left(q\right)>0\right)>0.9$). In contrast, specialist species predominantly showed lower genetic diversity than expected across their ranges ($P\left(\log\left(q\right)<0\right)>0.9$; Figure 6).

Edge populations in both Indian and Pacific regions consistently displayed lower genetic diversity than expected, regardless of host preference. This trend was observed in *A. clarkii* populations in the Maldives and Thailand, *A. akallopisos* in Madagascar, Mayotte and Kenya, *A. chrysopterus* in East Melanesia and Polynesia, and *A. melanopus* and *A. perideraion* in the Great Barrier Reef. Gulf of Thailand populations of *A. perideraion* and *A. polymnus* also exhibited reduced genetic diversity, probably due to the dramatic transformation of the area during the LGM, when extensive coastal areas were exposed as terrestrial landscapes inducing massive habitat loss. These patterns highlight the role of the Coral Triangle as a reservoir for genetic diversity, emphasizing its importance as a refugium during Quaternary sea level fluctuations, which hosts the highest concentration of suitable coral reef habitats (Pellissier et al. 2014).

*MAPS* analyses revealed that spatial patterns of population density closely mirror the patterns derived from *EEMS* for genetic diversity (Figure S7). However, both generalist and specialist species exhibited significant global declines in population densities from the LGM to recent times (Figure 4b, Figure S8). During the LGM (∼19,125 ya), specialists had significantly higher population densities than generalists (specialists: $231\pm 285\;N/km^{2}$; generalists: $27.6\pm 57.1\;N/km^{2}$; W=29, df=1, *p* < 0.001). These differences became nonsignificant following the rise of sea level (specialists: $0.002\pm 0.003\;N/km^{2}$; generalists: $0.01\pm 0.025\;N/km^{2}$; W=80, df=1, *p* = 0.075), indicating a more pronounced decline in the density of specialist populations during this transition. Additionally, specialists showed limited spatial variation in population densities both within and across species, whereas generalists exhibited stronger variation across geographic landscapes and more pronounced inter‐species differences.

## 4 Discussion

Pleistocene sea level fluctuations induced global population bottlenecks and structured populations across tropical marine taxa (Ludt and Rocha 2015). These fluctuations alternately fragmented and reconnected marine habitats, driving cycles of genetic differentiation and gene flow that varied between species according to their ecological characteristics (Blanco Gonzalez et al. 2016; Rönkä et al. 2021). Considering the obligate mutualism of clownfish with their host sea anemones, we hypothesized that generalist clownfish would generally maintain stable populations and connectivity. This stability is attributed to their greater adaptability to host environments and habitat connectivity. In contrast, specialists would suffer severe bottlenecks and greater reproductive isolation, constrained by specific host availability. This scenario would have lasting effects on current populations, potentially leaving specialists more vulnerable to new environmental threats.

Here, we show that Pleistocene sea‐level fluctuations differentially impacted clownfish demographic histories and current population structures based on their host specialisation. While generalist species maintained relatively stable $N_{e}$, all three specialist groups—*Radianthus*, *Entacamea* and *Stichodactyla* specialists—suffered significant and consistent $N_{e}$ declines, showing no signs of recovery with posterior sea level rise. Given the decreasing temporal resolution of MSMC estimates deeper in time, especially prior to 300 kya, the correlation between demographic declines and sea‐level regressions should be interpreted as approximate. Nonetheless, the consistent pattern across species and alignment with known intervals of habitat contraction support a biologically meaningful association. The substantial reductions of $N_{e}$ seen in specialists (42%–78%) and directly related to the drop in sea level underscore their acute sensitivity to habitat fragmentation, supporting our first hypothesis and providing empirical evidence for theoretical predictions on the increased susceptibility of ecological specialists to environmental fluctuations (Travis 2003).

Specialists species historically had higher $N_{e}$ than generalists. However, they experienced severe population bottlenecks that resulted in lower $N_{e}$ than generalists in the Holocene. This suggests that while specialisation may confer advantages during periods of environmental stability, it also increases vulnerability during climatic changes (Futuyma and Moreno 1988; Kassen 2002). Consequently, clownfish specialists struggled to recover $N_{e}$, even after sea level rise and habitat reconnection, putting them at increased risk of population decline and extinction (Büchi and Vuilleumier 2014; Reed and Tosh 2019). Our findings provide contemporary empirical support for patterns observed in the fossil record, corroborating the hypothesis that specialists experience disproportionately higher extinction rates in response to environmental perturbations (Clavel et al. 2011). This alignment between paleontological evidence and modern genomic data underscores the persistent vulnerability of ecological specialists to large‐scale environmental changes on evolutionary timescales.

Specialist $N_{e}$ declines indicate a limited adaptive potential of specialised species and greater vulnerability to environmental changes (Willi et al. 2006; Boulding 2008). It also likely contributed to the diversification of the clownfish clade during this epoch with allopatric speciation occurring predominantly among specialist species (Litsios et al. 2014; Gaboriau et al. 2018, 2025). The synchronous occurrence of diversification bursts and significant bottleneck events suggests that habitat fragmentation could have facilitated these speciation processes. In many other taxa, habitat fragmentation due to climatic changes or natural barriers has similarly driven allopatric speciation and diversification (Tolley et al. 2011; Dias et al. 2013). Nevertheless, we cannot disregard the importance of reproductive behaviour and dispersal abilities in driving clownfish speciation (Jones et al. 2005), particularly after geographical expansions of the late Pliocene and early Pleistocene (Gaboriau et al. 2025).

In addition, host anemones, like many other marine organisms, were also affected by Pleistocene sea level fluctuations (Ludt and Rocha 2015). Although specialist clownfish and their specific hosts likely experienced comparable declines (Chomicki et al. 2019), generalists may have benefited from their ability to exploit diverse hosts, thus avoiding being constrained by the demographics of a single host and, therefore, exhibiting greater survival (Kiers et al. 2010; Fontúrbel et al. 2021). An upward trend in $N_{e}$ in generalist populations about 100,000 years ago could be attributed to their opportunistic behaviour, exploiting habitats vacated by specialists undergoing bottlenecks. This phenomenon, observed in other taxa in response to climate change (Weiskopf et al. 2020), is also supported by the fossil record, indicating that past ecosystems shifted toward dominance by generalist species during such periods (Blois et al. 2013). However, the declining trends observed in Pacific populations of generalists *A. chrysopterus* and *A. clarkii* suggest that local factors can override the advantages of ecological generalism, and underscores the importance of considering both species‐level traits and local conditions when assessing population vulnerability (Nielsen et al. 2021; Andrew et al. 2022).

Our results show that sea level changes and ocean dynamics, especially tides and currents, were crucial in clownfish recolonization, highlighting the role of ocean currents in fish larval dispersal across fragmented habitats (Treml et al. 2008; Cowen and Sponaugle 2009; Morrison and Sandin 2011). As sea levels rose after LGM, both generalist and specialist clownfish species experienced increased dispersal distances, likely due to enhanced habitat connectivity, facilitating rapid marine recolonization (Hoarau et al. 2007; Jenkins et al. 2018; Boavida et al. 2019). The Indonesian Through‐Flow appears to have been the most important dispersal corridor in clownfish, facilitating stepping‐stone processes of genetic exchange between the Indian and Pacific Oceans. In contrast, clownfish dispersal is limited by geographical barriers such as the Sunda and Sahul shelves, shaped by glacial periods and the oceanic gaps in Melanesia and the Indian Ocean (White et al. 2010; Drew and Amatangelo 2017), presenting common geographical barriers to clownfish dispersal. These barriers, also characterised by strong tidal variations or current gyres (Connolly et al. 2003; Daryabor et al. 2016), contribute significantly to genetic isolation and differentiation between clownfish populations (Drew and Amatangelo 2017; Huyghe and Kochzius 2018; Snead et al. 2023).

Despite the common geographical characteristics shaping clownfish dispersal patterns, we found that host specialisation significantly influences population connectivity and genetic structure in clownfish, supporting the theoretical concept of ecological specialisation (Grosberg and Cunningham 2001; Louis et al. 2014; Selkoe et al. 2015). Generalist species demonstrate greater connectivity, particularly among core populations, while specialists encounter larger geographical barriers impeding gene flow and contributing to reproductive isolation. In particular, specialists exhibited greater dispersal distances compared to generalists, possibly as a compensatory mechanism to mitigate high levels of inbreeding and self‐recruitment (Clobert et al. 2009; Bonte et al. 2012; Lowe and McPeek 2014) through mechanisms such as extended pelagic larval phases (Shanks 2009).

Although specialists may have developed stronger dispersal abilities to adapt to historical conditions, the difference in dispersal distances between generalists and specialists is small (averaging less than 30 km), which is negligible considering the global distributions and the oceanographic scales that shape the biogeography of these species. We observed predominantly lower migration rates and connectivity among specialist populations than expected on the basis of geographical distance. These findings corroborate previous studies done on specific specialist clownfish that showed reduced genetic connectivity, particularly among populations that became isolated during the Pleistocene sea level lowstand (Timm and Kochzius 2008; Dohna et al. 2015; Huyghe and Kochzius 2017). Despite longer dispersal distances, specialists remain constrained by oceanographic barriers, possibly due to historical limitations in availability and/or fragmented distribution of suitable host anemones (Branconi et al. 2020).

Specialist clownfish face significant challenges in maintaining connectivity across habitats—a threat exacerbated by climate change and anthropogenic impacts—which reduce genetic diversity and reproductive success in contemporary populations (Hoegh‐Guldberg et al. 2007; Hughes et al. 2017; Saenz‐Agudelo et al. 2011; Madduppa et al. 2018; Moore, Jolly, et al. 2023). The observed differences in genetic diversity and population structure between generalists and specialists highlight the influence of ecological specialisation on evolutionary patterns. Specialist populations exhibited stronger genetic differentiation and lower diversity, consistent with historical bottlenecks, reduced gene flow and restricted dispersal—likely associated with narrow host preferences—highlighting their heightened extinction risk (Beldade et al. 2017; Moore, Jolly, et al. 2023). In contrast, generalists—occupying broader ecological niches—showed greater genetic connectivity, lower differentiation and slightly higher diversity, likely reflecting larger effective population sizes and more stable demographic histories (Zayed et al. 2005; Matthee 2020; Pasinelli 2022).

Although differences in *F*_ST_ between generalists and specialists were marginally non‐significant, generalists tended to show lower values, while specialists reached higher extremes, suggesting more consistent gene flow in generalists and greater isolation in specialists. However, it also reflects the effect of local dynamics. Isolated island populations may experience reduced connectivity or strong drift (Gaither et al. 2015; Young et al. 2018), elevating differentiation regardless of behavioural strategy. Such local processes may explain bottlenecks even in generalists (e.g., *A. clarkii* in the Maldives, *A. chrysopterus* in French Polynesia). Additionally, *F*_ST_ is sensitive to within‐population diversity (Delmore et al. 2018), sample size (Meirmans and Hedrick 2011) and effective population size (Lloyd et al. 2013), making cross‐species comparisons less reliable. Nonetheless, while $F_{\mathrm{ST}}$ would indicate genetic divergence driven by recent processes due to its sensitivity to recent gene flow and spatial structure (Slatkin 1987; Holsinger and Weir 2009), $d_{\mathrm{xy}}$ offers a more stable measure of absolute genetic divergence, capturing deeper evolutionary divergence (Delmore et al. 2018; Roux et al. 2016). Specialists consistently show higher absolute divergence in $d_{\mathrm{xy}}$, supported by a significant interaction with divergence time, indicating that long‐term isolation has played a stronger role in shaping their genomic landscape. Together, these metrics reveal how ecological specialisation, shaped by both behavioural and local demographic factors, drives contemporary and historical genetic differentiation.

Although none of the clownfish species are currently listed as endangered on the IUCN Red List, most lack sufficient population data to accurately assess their risk of extinction. Our findings suggest that specialist species are more vulnerable, underscoring the urgent need for a proper assessment and potentially targeted monitoring and conservation efforts. Mapping spatial patterns of genetic diversity and population density is critical for identifying at‐risk populations and prioritising actions such as habitat protection and restoration (Vucetich and Waite 2003; van Strien et al. 2014). Similarly, integrating genetic and ecological data can further improve predictions of resilience and adaptability, guiding more effective conservation strategies (Sommer et al. 2013; Keller et al. 2015).

Our findings place the Coral Triangle as a key reservoir of genetic diversity for clownfish. This region, which was a refugium during Quaternary sea level fluctuations (Pellissier et al. 2014), maintains greater intraspecific genetic diversity and $N_{e}$ compared to any other areas. The Coral Triangle constitutes a clownfish biodiversity hotspot, acting as the center of origin for the clade (Litsios et al. 2014) and harbouring the highest species diversity (Camp et al. 2016; García Jiménez et al. 2024). The patterns of genetic diversity observed mirror its biodiversity and probably result from the ecological complexity and environmental heterogeneity of its reef ecosystems (Pamilo 1988; Temunović et al. 2012; Abdul‐Rahman et al. 2021). Together, it underscores the vital role that this region plays in preserving the evolutionary potential of clownfish. In contrast, our results suggest that edge populations, characterised by reduced genetic diversity due to historical processes, geographic barriers and limited gene flow (Allendorf et al. 2012; Jamieson 2011), may require further assessment. Targeted conservation strategies may be needed to support their long‐term resilience and facilitate local adaptation (Frankham 2005; Szűcs et al. 2017).

## 5 Conclusion

Our study underscores the critical role of ecological specialisation in shaping the responses of the clownfish population to Pleistocene sea level fluctuations and the resulting habitat fragmentation. Generalist species, with their broader ecological niches, exhibited higher gene flow, genetic diversity and population stability, demonstrating resilience to environmental changes. In contrast, specialists faced reproductive isolation, severe bottlenecks and reduced genetic diversity, increasing their vulnerability to both historical and contemporary environmental challenges. Our findings add new empirical evidence to the theory of ecological specialisation by illustrating how narrower niches can drive divergent population dynamics and increase extinction risks for specialist species.

Our results connect past processes with current patterns to highlight vulnerable populations and the need for improved conservation assessments. Future research should build on these findings to refine conservation strategies and assess the impacts of climate change on clownfish and their hosts, crucial to predicting and mitigating challenges for these iconic reef fish. Such efforts will not only enhance the effectiveness of conservation strategies, but also contribute to broaden our understanding of the resilience and adaptability of marine populations in the face of accelerating global change.

### Biological Sampling Permits

The research permits obtained for this study are the following

**Table:** 

Country	Permit number
Mayotte	06/UTM/2016
New Caledonia	N°60912‐895‐2017/JJC
Kenya	NCST/RRI/12/1/BS/250
Indonesia	55/PSTK/UH/XI/04
Thailand	0401/16454
Madagascar	Samples collected between 2012 and 2015, before the implementation of Nagoya protocol by national authorities. Sampling was approved by and under the supervision of the ‘Institut Halieutique et des Sciences Marines’ of Toliara.
Australia	
Queensland Department of Primary Industries	118636, 150981
QLD General Fisheries Permit:	180731
Great Barrier Marine Park (GBRMPA)	G17/38160.1
Queensland Parks and Wildlife Marine Parks	G08/26733.1
	G08/28114.1
	G09/31678.1
	G10/33597.1
	G11/34452.1
	G11/34640.1
University of Queensland, Animal Ethics	QBI/304/16
New Caledonia	
Environment Direction of the South Province	N°60912‐895‐2017/JJC
Environment Direction of the North Province	N°1291‐2017/ARR/DENV
Maldives	OTHR30‐D/INDIV/2016/538
Solomon Islands	Permit to S. Albert
Papua New Guinea	Research Visa: 10350008304
	Permit to Export Wildlife: 011318
Taiwan	03/F/0390B
Philippines	
Bureau of Fisheries and Aquatic Resources	Commodity Clearance 2016‐20091
Palawan Council for Sustainable Development (PCSD)	GP 2016‐03, Wildlife Transport Permit no. 2016‐05‐000062‐DMO–Calamianes
Fisheries inspection and quarantine clearance	009391

## Author Contributions

Alberto García Jiménez, Théo Gaboriau and Nicolas Salamin conceptualised and designed the study. Alberto García Jiménez conducted the research, performed the analyses, interpreted the results and wrote the manuscript. Fieldwork expeditions and sample collection were carried out by Alberto García Jiménez, Théo Gaboriau, Lucy M. Fitzgerald, Sarah Schmid, Anna Marcionetti, Sarah Schmid, Joris Bertrand, Abigail Shaughnessy, Carl Santiago, Bruno Frédérich, Fabio Cortesi, Ploypallin Rangseethampanya, Phurinat Ruttanachuchote, Wiphawan Aunkhongthong, Sittiporn Pengsakun, Makamas Sutthacheep and Thamasak Yeemin. Marion Talbi and Milan Malinsky generated the recombination maps. Théo Gaboriau and Nicolas Salamin contributed to data interpretation and manuscript writing. All authors reviewed, revised and approved the final version of the manuscript.

## Disclosure

Benefits‐Sharing Statement: The sharing of our data and results on public databases ensures transparency and facilitates further research in the field. All fieldwork conducted during this study adhered to local regulations and was carried out in collaboration with local entities, including the University of Mayotte (Mayotte), University of New Caledonia (New Caledonia), Kenya Marine and Fisheries Research Institute (Kenya), National Research Institute (Papua New Guinea), Universitas Hasanuddin (Indonesia), University of Ramkhamhaeng (Thailand), University of Toliara and the Ministry of Marine Resources and Fisheries (Madagascar), Lizard Island Research Station and University of Queensland (Australia). Our research activities were conducted in accordance with the “Access and Benefit Sharing” (ABS) principles of the Nagoya Protocol established by the Convention on Biological Diversity (CBD).

Samples from Indonesia (2013), Maldives (2017), New Caledonia (2017), Thailand (2022), Madagascar (2022), and Australia (2023) were obtained by Nicolas Salamin's research group. Samples from Solomon Islands, Papua New Guinea, and Lizard Island (2018) were obtained by Cynthia Riginos' research group. Sampling in the Solomon Islands was via the Australian Government's Pacific Strategy Assistance Program and with the assistance of the Roviana Conservation Foundation. Samples from Taiwan were obtained by Marc Kochzius. Samples from the Philippines were obtained by Jimmy O'Donnell.

## Conflicts of Interest

The authors declare no conflicts of interest.

## Supporting information


**Data S1:** mec70134‐sup‐0001‐DataS1.zip.

## Data Availability

Data and scripts with instructions to reproduce the analysis and figures can be found at https://github.com/agarciaEE/ClownfishPopGen_GarciaJimenez_etal2024 (DOI: 10.5281/zenodo.14387999). Genomic data in the form of raw reads has been deposited in the NCBI Sequence Read Archive (SRA), with new data under the bioproject ID: PRJNA1202426. All sample information can be found in the samples_info.csv file provided in the repository. Access to these data will also be provided upon acceptance of the manuscript.
