# Supplementary material for: Habitat Specialisation Impacts Clownfish Demographic Resilience to Pleistocene Sea‐Level Fluctuations
Source: Mol Ecol. 2025 Oct 13;34(22):e70134. doi: 10.1111/mec.70134 (PMC12617074; doi:10.1111/mec.70134)
Supplement: Supplementary file 1 — Data S1: mec70134‐sup‐0001‐DataS1.zip. [file MEC-34-e70134-s001.zip › Habitat_Specialization_Impacts_Clownfish_Resilience_R1_SUPP.pdf]

Habitat specialization impacts clownfish demographic resilience to Pleistocene sea-Level fluctuations

Alberto García Jiménez[1*], Marion Talbi[2], Lucy M. Fitzgerald[1], A Heim[1], Anna Marcionetti[1], Sarah Schmid[3], Joris Bertrand[4], Abigail Shaughnessy[7], Carl Santiago[7], Ploypallin Rangseethampanya[5], Phurinat Ruttanachuchote[5], Wiphawan Aunkhongthong[5], Sittiporn Pengsakun[5], Makamas Sutthacheep[5], Milan Malinsky[2], Bruno Frédérich[6], Fabio Cortesi[7], Marc Kochzius[8], Thamasak Yeemin[5], Théo Gaboriau[1], Nicolas Salamin[1]

[1]Department of Computational Biology, University of Lausanne, Lausanne, Switzerland.
[2]Department of Aquatic Ecology & Evolution, University of Bern, Bern, Switzerland.
[3]Department of Environmental Systems Science, ETH Zürich, Zürich, Switzerland.
[4]Laboratoire Génome et Développement des Plantes, UPVD, Perpignan, France.
[5]Marine Biodiversity Research Group, Ramkhamhaeng University, Bangkok, Thailand.
[6]Laboratory of Evolutionary Ecology, FOCUS, University of Liège, Liège, Belgium.
[7]Queensland Brain Institute, The University of Queensland, Brisbane, Australia.
[8]Marine Biology - Ecology, Evolution and Genetics (bDIV), Vrije Universiteit (VUB), Brussels, Belgium.

*Corresponding author(s). E-mail(s): agarcia26286@gmail.com;

Abstract

Habitat fragmentation and loss are key threats to biodiversity, yet their impacts on marine species remain poorly understood. Clownfishes, which rely on sea anemones for shelter and reproduction, provide an interesting model to explore how ecological specialization mediates species responses to habitat perturbations. We used whole-genome data from 382 individuals across ten species with varying host specializations to reconstruct demographic histories and infer spatial genetic structure to assess the impact of Pleistocene sea-level fluctuations. Generalist species, associated with multiple hosts, maintained stable effective population sizes ($\boldsymbol{N_e}$) and population connectivity during habitat fragmentation, reflecting resilience to environmental instability. In contrast, specialists experienced severe $\boldsymbol{N_e}$ declines and genetic structuring, driven by their dependence on specific hosts, without signs of population recovery following habitat reconnection. Spatial genomic analyses identified the Indonesian Through-Flow as a key dispersal corridor and the Coral Triangle as a critical hub of genetic diversity, while continental shelves and extensive open ocean regions appeared as barriers to gene flow. Our findings reveal how host specialization shapes clownfish population dynamics, emphasizing the importance of incorporating ecological dependencies into conservation assessments and deepening our understanding of species responses to ecological constraints and environmental changes over evolutionary timescales.

Keywords: Clownfish Mutualism, Habitat Specialization, Sea-Level Fluctuations, Demographic Resilience, Population Genomics, Genetic Structure

Supplementary Figures

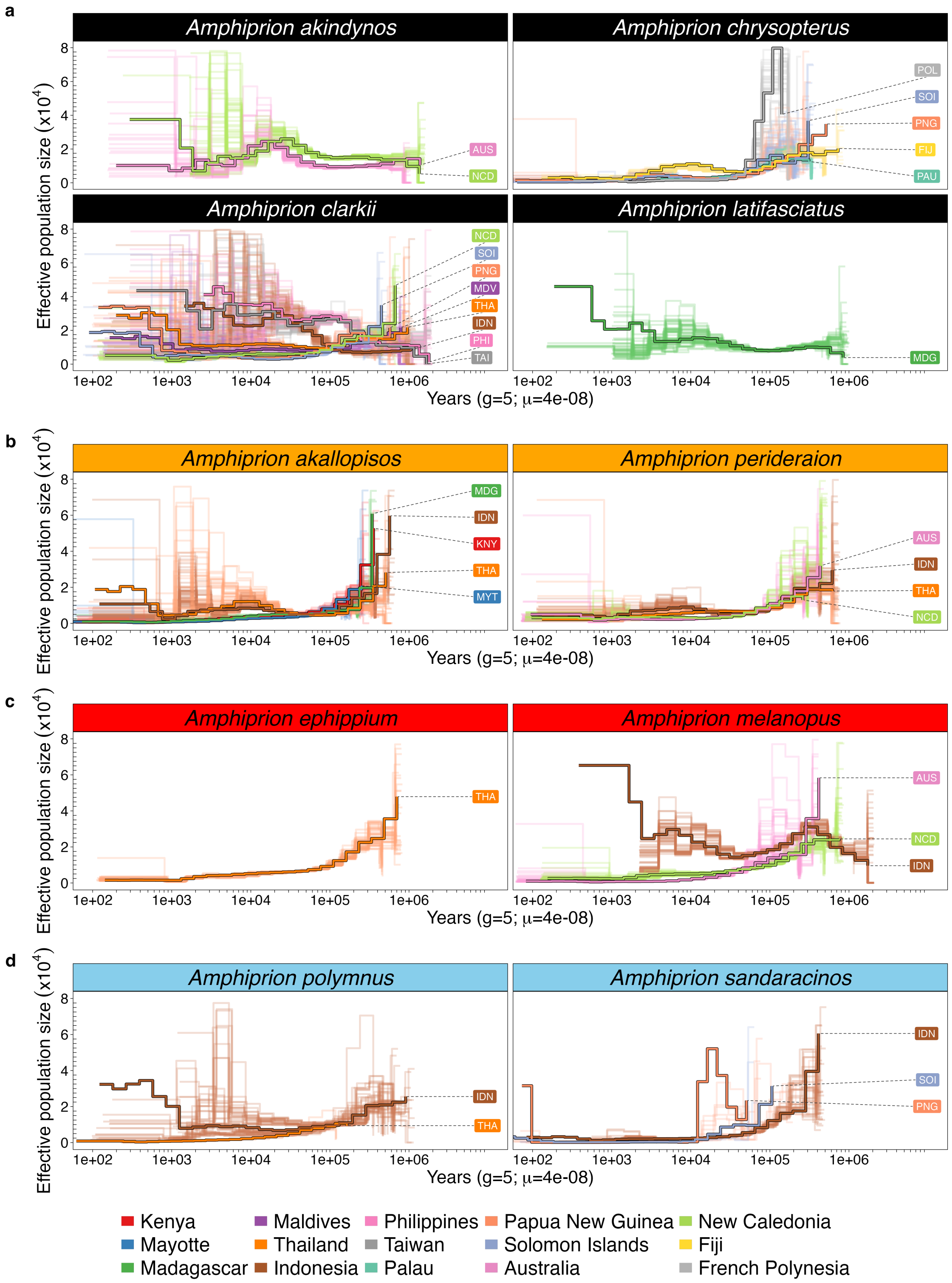

Fig. 1: **Demographic reconstructions of all populations of each species**. Thick colored line shows the average trajectory over ten msmc runs using 3 random individuals each time from each population. Light colored lines represent 50 bootstraps on each population using msmc-tools function 'multihetsep_bootstrap.py'. a) Generalists; b) *Radianthus magnifica* specialists (RM specialists); c) *Entacmaea quadricolor* specialists (EQ specialists); and d) *Stichodactyla* specialists (SD specialists).

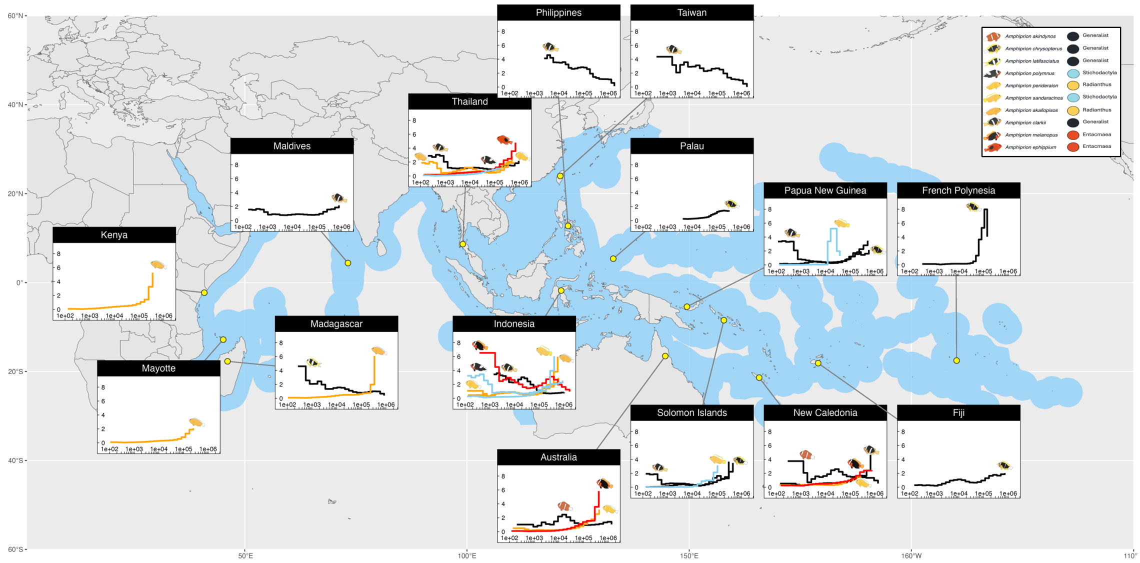

Fig. 2: **Demographic trajectories per geographic location.** Distribution map of clownfishes shown in light blue polygons over the Indo-Pacific with species MSMC reconstructions of each population (yellow filled-circle). Names of the country where the population was sampled is indicated at the top of each msmc plot. N_e reconstruction lines are colored accordingly to the host specialization (black for Generalists, orange for *Radianthus* specialists, red for *Entacmaea* specialists, and blue for *Stichodactyla* specialists.). N_e values are in the scale of 10^4 and time axis shows a time frame from 10^3 to 10^6 years ago considering a mutation rate (μ) of $4e^{-8}$ and a generation time of 5 years.

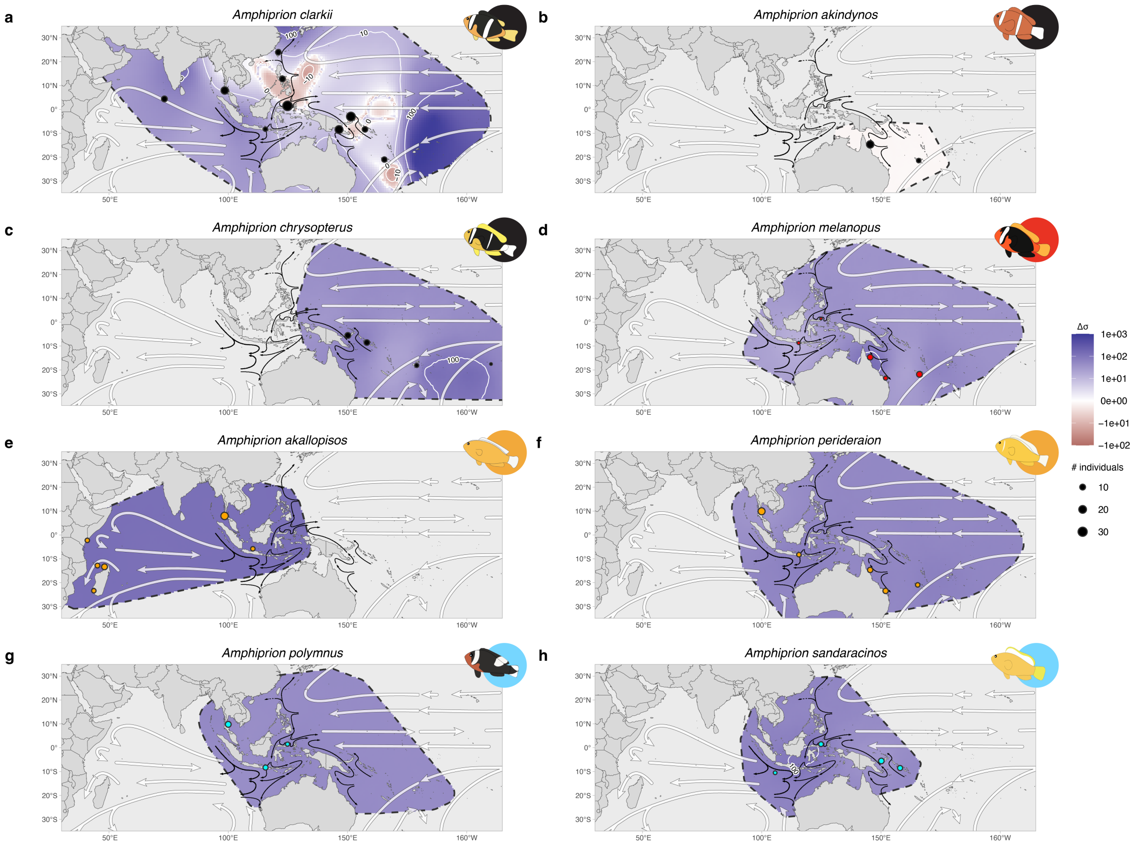

Fig. 3: **Temporal changes of dispersal between the LGM and present day.** Panels (a-h) show the difference on dispersal distances per generation ($\Delta\sigma$) between ∼20,000 and ∼62.5 years ago for eight clownfish species. The maps show the distribution range of the clownfish clade. The dashed polygon indicates the area used for inference, based on a 1,000 km buffer around the species distributions. Sampling locations are represented by circles, with size indicating the number of individuals sampled and color denoting host anemone specialization (black: generalist species; orange: *Radianthus* specialists; red: *Entacmaea* specialists; blue: *Stichodactyla* specialists). White arrows indicate global oceanic currents, while dark arrows show Coral Triangle currents. The color gradient represents the difference in σ values (in km) between present day and LGM, with blue indicating an increase and red a decrease in dispersal distance. Species names are displayed at the top of each map, with vector illustrations of each species and their corresponding host anemone category shown in the top-right corner.

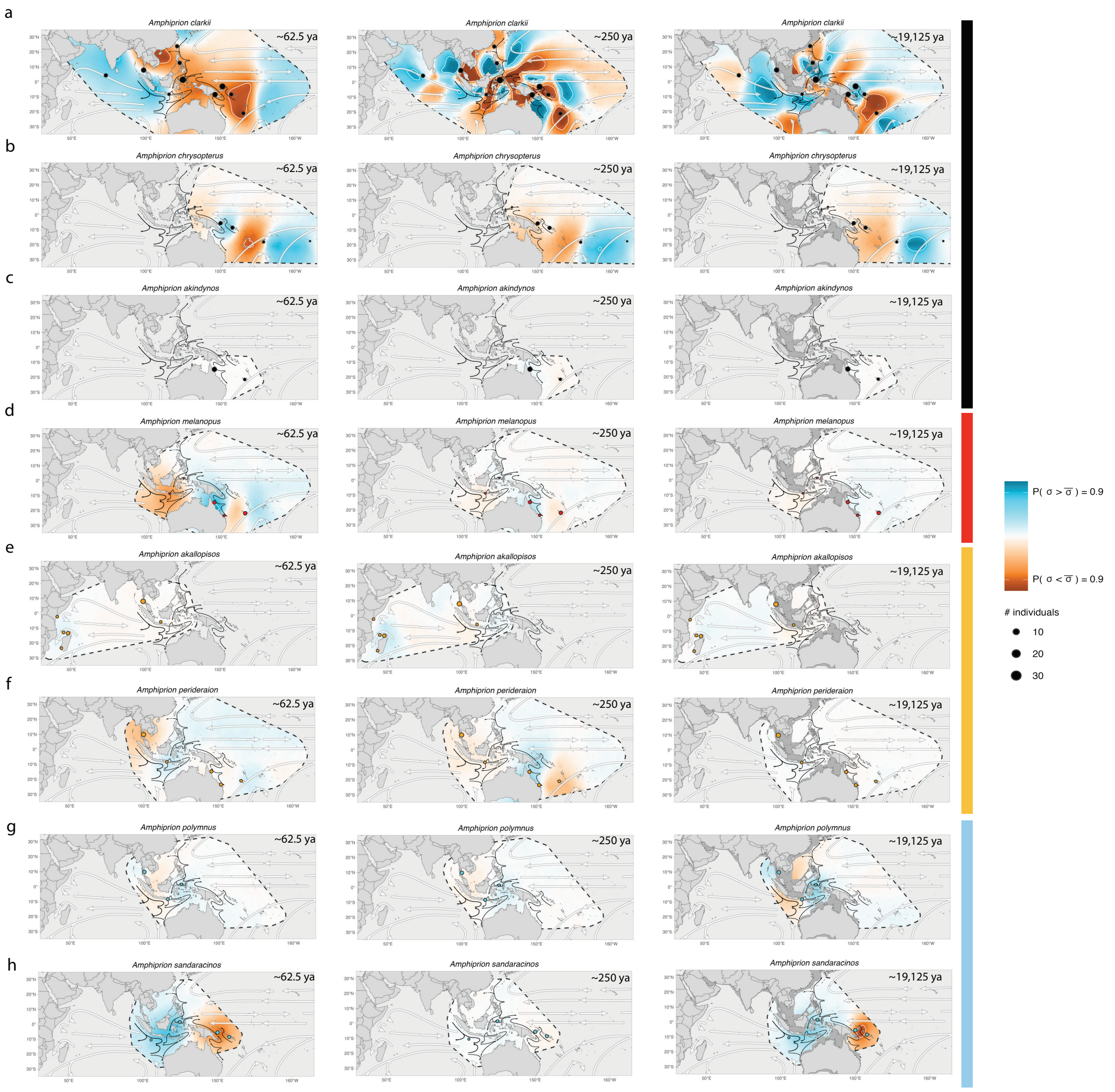

Fig. 4: **Spatial patterns of dispersal distances.** Probabilities of higher or lower than average dispersal distance for eight species (a–h) across three time periods (∼62.5 years ago, ∼250 years ago, and ∼19,125 years ago). Each panel shows MAPS maps of the probability that dispersal distances at a given location are higher or lower than the average across the species' range. Darker blue areas indicate regions where population density is significantly higher than average ($P(\sigma > \bar{\sigma}) > 0.9$), while darker red areas indicate regions with significantly lower ($P(\sigma < \bar{\sigma}) > 0.9$) population densities (see legend on the right). Areas of significance are outlined with white contour lines in the maps. Land masses are shown in grey, with darker grey areas in the oldest time period (∼19,125 years ago, LGM) indicating regions that were above sea level at the time. White arrows indicate global oceanic currents, while dark arrows show Coral Triangle currents. The dashed polygons outline the minimum convex polygon area encapsulating each species' distribution used in MAPS analyses. Species names are displayed at the top of each plot, with the corresponding time period shown in the top-right corner of the panels, arranged chronologically from left (most recent) to right (LGM period). Sample locations are shown as circles, whose sizes correspond to sample size (see legend), and their colors reflect host preferences: black for generalists, orange for RM specialists, red for EQ specialists, and light blue for SD specialists. Additionally, colored bars to the right of the figure indicate species categories, matching the circle color scheme.

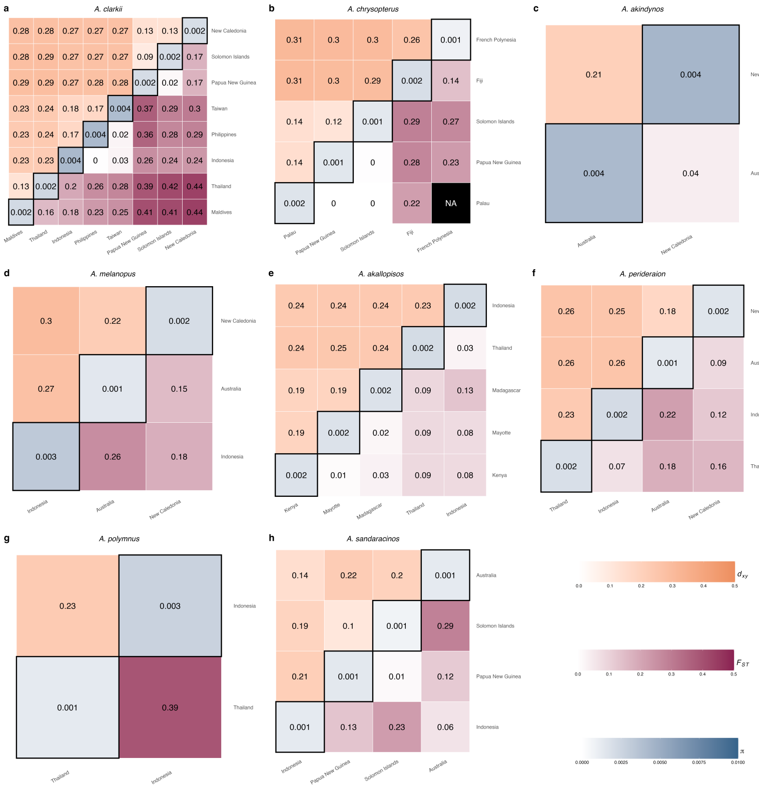

Fig. 5: **Genetic diversity and differentiation across clownfish species.** Panels (a-h) show pairwise dxy (upper diagonal), F_{ST} (lower diagonal) and nucleotide diversity (π; diagonal) between populations of ten clownfish species. Heatmaps are color-coded from white to muted orange for dxy, white to deep steel blue for F_{ST}, and white to dark violet-red for π (see legend at bottom-left). Species names are listed at the top of each panel. Values are indicated inside each cell. Species names are shown at the top of each panel.

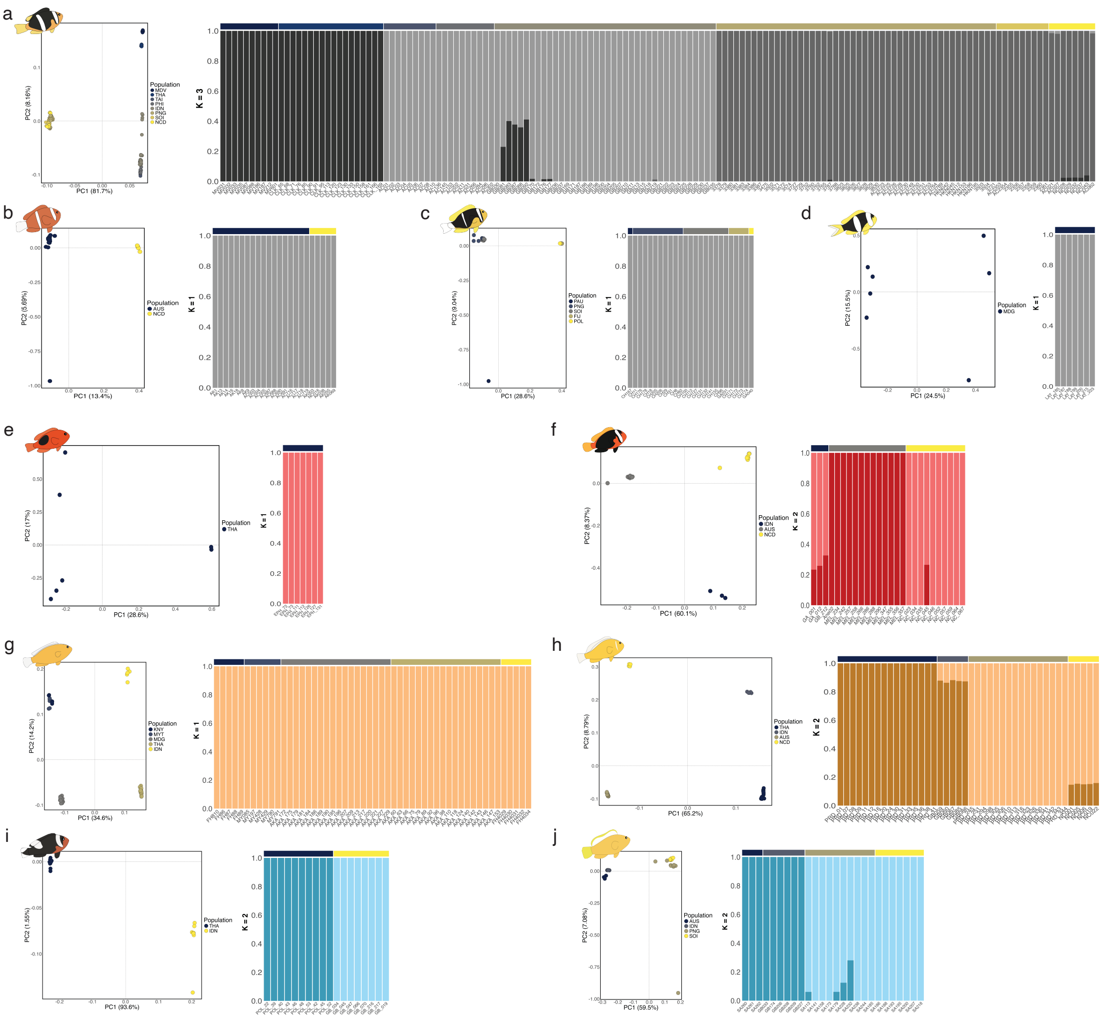

Fig. 6: **Population structure of the 10 clownfish species.** Color codes are according to their host regime (black for generalists, red for EQ specialists, orange for RM specialists and blue for SD specialists). For each species, left-panel shows the first and second principal components of the PCA. Proportion of variance explain for each axis is indicated at the axis label between parenthesis. Colored points are according to the population (see legend); and right-panel shows ADMIXTURE plot of the best K, i.e. the lowest cross-validation error. Colored bars at the top of each admixture plot indicate different populations according to PCA's legend. Bar heights show the proportion of each individual's genome assigned to the color-coded genetic clusters. Vector illustrations of each species and their corresponding host anemone category shown in the top-left corner. a) *A. clarkii*, b) *A. akindynos*, c) *A. chrysopterus*, d) *A. latifasciatus*, e) *A. ephippium*, f) *A. melanopus*, g) *A. akallopisos*, h) *A. perideraion*, i) *A. polymnus*, and j) *A. sandaracinos*.

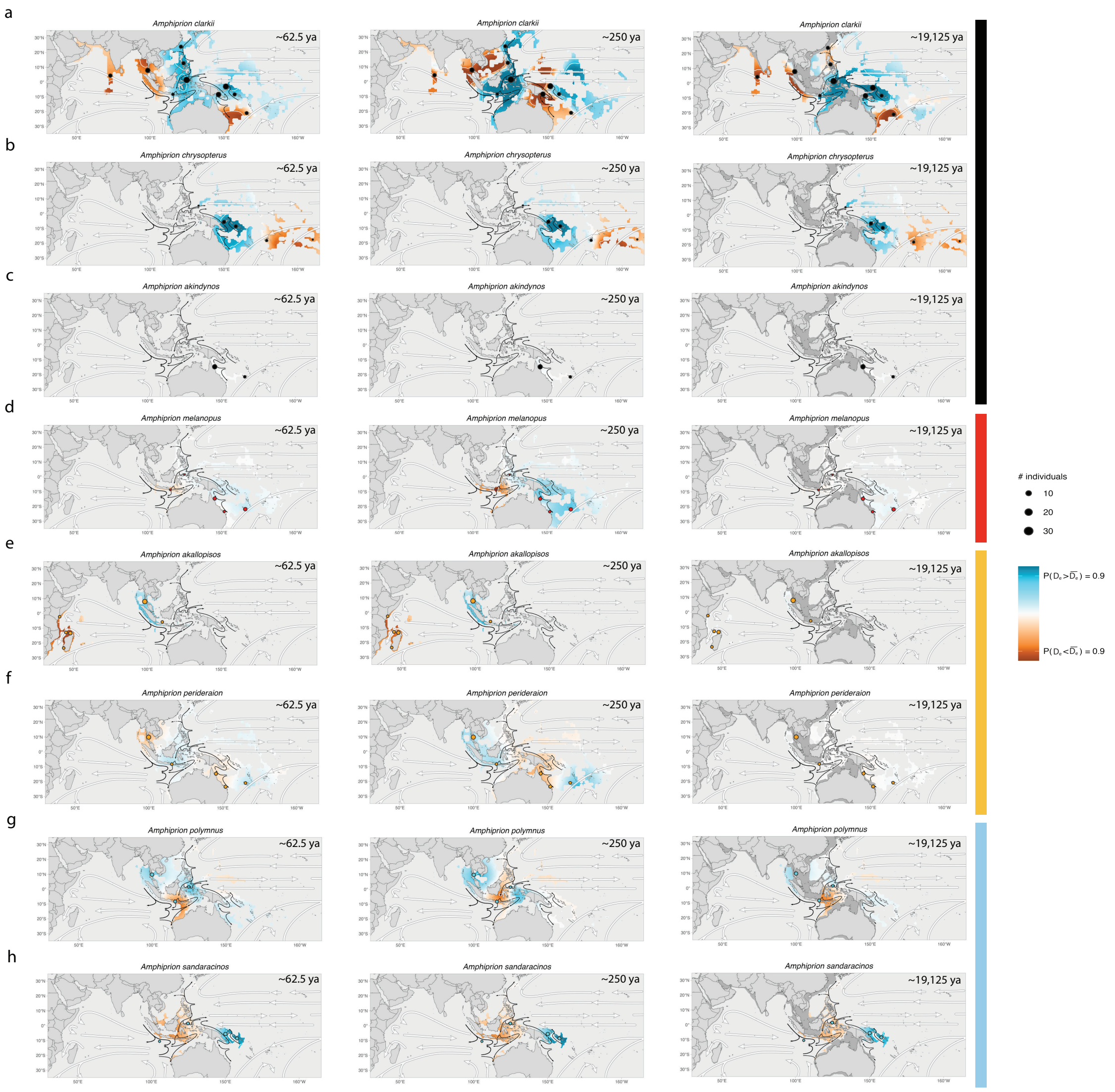

Fig. 7: **Spatial patterns of population density** Probabilities of higher or lower than average population density for eight species (a–h) across three time periods (∼62.5 years ago, ∼250 years ago, and ∼19,125 years ago). Each panel shows *MAPS* spatial inference of the probability that population density at a given location is higher than the average across the species' range. Darker blue areas indicate regions where population density is significantly higher than average ($P(D_e > \bar{D}_e) > 0.9$), while darker red areas indicate regions with significantly lower ($P(D_e < \bar{D}_e) > 0.9$) population densities (see legend on the right). Areas of significance are outlined with white contour lines in the maps. Land masses are shown in grey, with darker grey areas in the oldest time period (∼19,125 years ago, LGM) indicating regions that were above sea level at the time. White arrows indicate global oceanic currents, while dark arrows show Coral Triangle currents. Maps are masked by species' inferred distributions derived from *ENMTML* models. Species names are displayed at the top of each plot, while the time period corresponding to each map is shown in the top-right corner, ordered chronologically from left (most recent) to right (LGM period). Sample locations are represented by circles, whose sizes correspond to the sample size (see legend). The circles are colored to indicate host preferences: black for generalists, orange for RM specialists, red for EQ specialists, and light blue for SD specialists. Additionally, colored bars to the right of the figure indicate species categories based on mutualistic behavior, matching the circle color scheme.

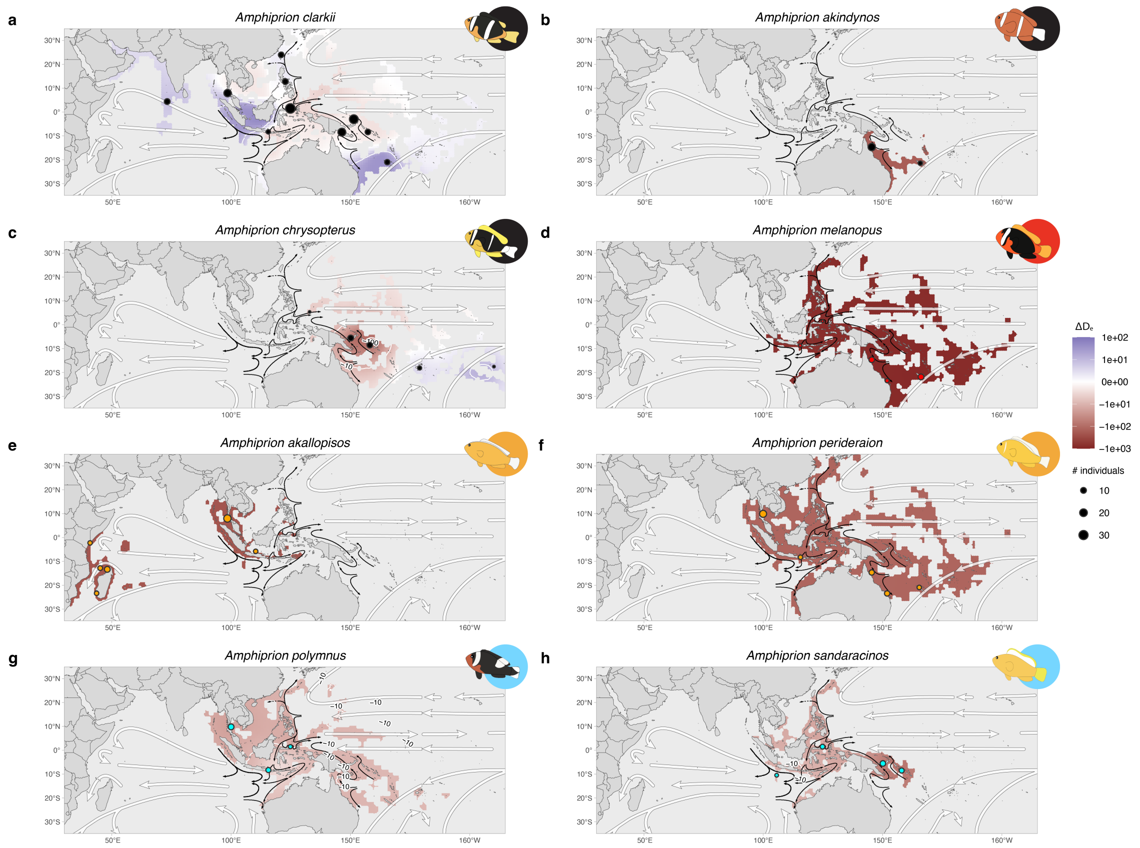

Fig. 8: Comparison of spatial patterns in population density between the Last Glacial Maximum (LGM, ∼20,000 years ago) and present day (∼62.5 years ago) for eight clownfish species, estimated using MAPS (**?**). The maps show the distribution range of the clownfish clade. Sampling locations are represented by circles, with size indicating the number of individuals sampled and color denoting host anemone specialization (black: generalist species; orange: *Radianthus* specialists; red: *Entacmaea* specialists; blue: *Stichodactyla* specialists). White arrows indicate global oceanic currents, while dark arrows show Coral Triangle currents. The color gradient represents the difference in population density (individuals per km^2) between present day and LGM, with blue indicating an increase and red a decrease in population density. Estimated values outside the inferred species distribution using ENMTML (**?**) are masked. Species names are displayed at the top of each map, with vector illustrations of each species and their corresponding host anemone category shown in the top-right corner.

Supplementary Tables

Table 1: Summary of Fixed Effects from Generalized Linear Mixed Model (GLMM) examining the relationship between sea level fluctuations, host categories, and effective population size (N_e). Estimates are provided with 95% confidence intervals (CI), and statistical significance is denoted by asterisks.

term	estimate	conf.low	conf.high	p.value	significance
(Intercept)	-0.035	-0.618	0.548	0.906	
seaLevel	-0.036	-0.067	-0.006	0.018	*
time	-0.015	-0.048	0.018	0.367234	
RM specialist	-0.348	-0.628	-0.067	0.015	*
EQ specialist	-0.153	-0.483	0.176	0.361	
SD specialist	-0.321	-0.630	-0.012	0.042	*
Mayotte	-0.216	-0.940	0.508	0.558	
Madagascar	0.002	-0.641	0.644	0.995	
Maldives	0.110	-0.666	0.886	0.781	
Thailand	0.166	-0.414	0.746	0.575	
Indonesia	0.660	0.082	1.239	0.025	*
Philippines	0.878	0.102	1.654	0.026	*
Taiwan	0.805	0.029	1.581	0.042	*
Palau	-0.265	-1.042	0.513	0.504	
Papua New Guinea	0.275	-0.377	0.928	0.408	
Solomon Islands	-0.197	-0.843	0.449	0.550	
Australia	0.122	-0.493	0.738	0.696	
New Caledonia	0.133	-0.470	0.736	0.664	
Fiji	0.069	-0.707	0.845	0.861	
French Polynesia	0.110	-0.667	0.887	0.781	
seaLevel:RM specialist	-0.023	-0.083	0.037	0.446	
seaLevel:EQ specialist	-0.133	-0.201	-0.065	< 0.001	***
seaLevel:SD specialist	-0.155	-0.245	-0.064	< 0.001	***
seaLevel:time	-0.190	-0.236	-0.144	< 0.001	***
RM specialist:time	1.315	1.222	1.408	< 0.001	***
EQ specialist:time	0.439	0.366	0.512	< 0.001	***
SD specialist:time	1.153	0.987	1.319	< 0.001	***
seaLevel:RM specialist:time	0.426	0.310	0.541	< 0.001	***
seaLevel:EQ specialist:time	-0.135	-0.237	-0.033	0.009	**
seaLevel:SD specialist:time	0.689	0.515	0.863	< 0.001	***

Table 2: Summary of the Linear Mixed Model (LMM) analysis for F_{ST} (a) and d_{xy} (b) as a function of geographical distance, divergence time, and mutualistic behavior (Specialist vs. Generalist). Significant predictors ($p < 0.05$) are marked with asterisks; marginally significant predictors ($p < 0.1$) are marked with a dot. Interaction terms indicate that the effect of spatial and temporal separation on genetic differentiation is modulated by behavioral specialization. Significance codes: * $p < 0.05$, ** $p < 0.01$, *** $p < 0.001$, **.** $p < 0.1$.

(a) F_{ST}

**Predictors**	**Estimates**	**95% CI**	***p***
Intercept	0.17	[+0.07, +0.28]	0.002 **
geographical distance	0.05	[+0.00, +0.10]	0.049 *
divergence time	0.07	[+0.02, +0.13]	0.005 **
behavior Specialist	0.18	[–0.01, +0.35]	0.052 **.**
geographical distance:divergence time	–0.00	[–0.03, +0.03]	0.967
geographical distance:behavior Specialist	–0.27	[–0.44, –0.10]	0.003 **
divergence time:behavior Specialist	0.12	[–0.04, +0.29]	0.127
geographical distance:divergence time:behavior Specialist	–0.26	[–0.44, –0.07]	0.007 **
**Random Effects**			
Residual variance (σ^2)	0.00		
Pop1 variance ($\tau_{00\ ppo1}$)	0.00		
Pop2 variance ($\tau_{00\ pop2}$)	0.00		
Species variance ($\tau_{00\ species}$)	0.01		
Intraclass Correlation Coefficient (ICC)	0.79		
**Model Fit**			
Marginal R^2	0.468		
Conditional R^2	0.890		
**Observations**			
Total	64		
**Number of Groups**			
N_{pop1}	13		
N_{pop2}	12		
$N_{species}$	8		

(b) d_{xy}

**Predictors**	**Estimates**	**95% CI**	***p***
Intercept	0.25	[+0.19, +0.30]	<0.001 ***
geographical distance	0.03	[+0.01, +0.05]	0.004 **
divergence time	0.04	[+0.02, +0.06]	<0.001 ***
behavior Specialist	0.08	[–0.00, +0.15]	0.053 **.**
geographical distance:divergence time	–0.00	[–0.02, +0.01]	0.658
geographical distance:behavior Specialist	–0.03	[–0.08, +0.03]	0.382
divergence time:behavior Specialist	0.06	[+0.01, +0.12]	0.022 *
geographical distance:divergence time:behavior Specialist	–0.01	[–0.07, +0.05]	0.753
**Random Effects**			
Residual variance (σ^2)	0.00		
Pop1 variance ($\tau_{00\ pop1}$)	0.00		
Pop2 variance ($\tau_{00\ pop2}$)	0.00		
Species variance ($\tau_{00\ species}$)	0.00		
Intraclass Correlation Coefficient (ICC)	0.93		
**Model Fit**			
Marginal R^2	0.517		
Conditional R^2	0.965		
**Observations**			
Total	65		
**Number of Groups**			
N_{pop1}	13		
N_{pop2}	12		
$N_{species}$	8		
